# Differential effects of warming on the complexity and stability of the microbial network in *Phragmites australis* and *Spartina alterniflora* wetlands in Yancheng, Jiangsu Province, China

**DOI:** 10.3389/fmicb.2024.1347821

**Published:** 2024-03-27

**Authors:** Lixin Pei, Siyuan Ye, Liujuan Xie, Pan Zhou, Lei He, Shixiong Yang, Xigui Ding, Hongming Yuan, Tianjiao Dai, Edward A. Laws

**Affiliations:** ^1^Qingdao Institute of Marine Geology, China Geologic Survey, Qingdao, China; ^2^Laboratory for Marine Geology, Qingdao Marine Science and Technology Center, Qingdao, China; ^3^School of Water Resources and Environment, China University of Geosciences (Beijing), Beijing, China; ^4^Department of Environmental Sciences, College of the Coast and Environment, Louisiana State University, Baton Rouge, LA, United States

**Keywords:** coastal wetland, climate change, microbial network, microorganism, carbon sequestration

## Abstract

The impact of climate warming on soil microbial communities can significantly influence the global carbon cycle. Coastal wetlands, in particular, are susceptible to changes in soil microbial community structure due to climate warming and the presence of invasive plant species. However, there is limited knowledge about how native and invasive plant wetland soil microbes differ in their response to warming. In this study, we investigated the temporal dynamics of soil microbes (prokaryotes and fungi) under experimental warming in two coastal wetlands dominated by native *Phragmites australis* (*P. australis*) and invasive *Spartina alterniflora* (*S. alterniflora*). Our research indicated that short-term warming had minimal effects on microbial abundance, diversity, and composition. However, it did accelerate the succession of soil microbial communities, with potentially greater impacts on fungi than prokaryotes. Furthermore, in the *S. alterniflora* wetland, experimental warming notably increased the complexity and connectivity of the microbial networks. While in the *P. australis* wetland, it decreased these factors. Analysis of robustness showed that experimental warming stabilized the co-occurrence network of the microbial community in the *P. australis* wetland, but destabilized it in the *S. alterniflora* wetland. Additionally, the functional prediction analysis using the Faprotax and FunGuild databases revealed that the *S. alterniflora* wetland had a higher proportion of saprotrophic fungi and prokaryotic OTUs involved in carbon degradation (*p* < 0.05). With warming treatments, there was an increasing trend in the proportion of prokaryotic OTUs involved in carbon degradation, particularly in the *S. alterniflora* wetland. Therefore, it is crucial to protect native *P. australis* wetlands from *S. alterniflora* invasion to mitigate carbon emissions and preserve the health of coastal wetland ecosystems under future climate warming in China.

## 1 Introduction

Coastal wetlands are important blue carbon ecosystems that regulate carbon sequestration, support biodiversity, and enhance ecosystem productivity globally (McLeod et al., 2011). However, they are highly vulnerable to climate change and human activities because of their location in intertidal zones (Osland et al., 2016; Schuerch et al., 2018). Among these threats, plant invasion and warming are particularly concerning threats. *Spartina alterniflora* (*S. alterniflora*), a globally invasive species, has spread from its native habitat along the Atlantic coast of North America to coastal areas in Asia, Europe, Oceania, southern Africa, and the Pacific coast of the United States over the past two centuries (Wang et al., 2006). Researches have shown that the invasion of *S. alterniflora* has changed the community structure of soil microorganisms, which in turn affects the carbon and nitrogen cycling processes in ecosystems (Yuan et al., 2016; Gao et al., 2022). For example, a study in Yancheng, Jiangsu Province, China, found that the invasion of *S. alterniflora* increased the abundance of soil ammonia-oxidizing archaea, methanogens, and sulfate-reducing bacteria, leading to accelerated soil carbon and nitrogen mineralization compared to native soil habitats (Yuan et al., 2016). Gao et al. (2022) discovered that invasion of *S. alterniflora* resulted in important reductions in the complexity and stability of microbial networks, decoupling the associations between microbes and carbon pools. While the influences of plant invasions on soil microbial communities and functions are well-documented (Lin et al., 2021; Gao et al., 2022; Hussain et al., 2023), there is limited knowledge about the influence of climate warming on soil microbes in coastal wetlands (Song et al., 2020). Specifically, it is unclear whether native and invasive plant wetlands respond differently to climate warming. Soil microbes are essential in the carbon cycle by influencing carbon mineralization and stabilization (Bardgett et al., 2008; Zhou et al., 2012). Changes in soil microbial communities can significantly impact greenhouse gas emissions from the soil (Davidson and Janssens, 2006). Therefore, studying the differential response of soil microbes in invasive and native plant wetlands to global warming can provide a more accurate assessment of the adaptability and stability of coastal wetland ecosystems under future climate change. This research can also serve as a scientific basis for projecting future carbon sequestration in coastal wetlands and developing effective management strategies.

Most studies on the impact of climate warming on ecosystems have focused on changes in microbial communities after several years warming (Xue et al., 2016; Song et al., 2020; Zhou S.-Y.-D. et al., 2023). Yet, little attention has been given to the impact of climate warming on the temporal succession of microbial communities. The succession can depend on factors such as spatial and temporal scales, ecosystem characteristics, types of disturbances, and functional traits (Zhou et al., 2014; Li et al., 2016). Previous research has shown that climate warming accelerates the turnover rates of soil microbial communities in grassland ecosystem, leading to a divergent succession of microbial communities (Guo et al., 2018). Understanding the dynamics of microbial communities over time is crucial for predicting how ecosystem stability, functionality, and services will be affected by climate warming (Prach and Walker, 2011). Therefore, to determine whether there are differences in the response of soil microbial communities in wetlands invaded by plants versus native plant wetlands to global warming, it is necessary to consider the temporal succession of microbial communities in addition to the conventional comparison of community structures.

Within soil ecosystems, microbial species interact with one another through mechanisms such as commensalism, cooperation, mutualism, competition, and predation (Faust and Raes, 2012; Banerjee et al., 2016). Such complex ecological relationships can be illustrated as networks that are able to explain the co-variation of species abundances (Faust and Raes, 2012; Pržulj and Malod-Dognin, 2016). Recent studies have demonstrated that changes in the structure of microbial networks in forest, grassland, and agricultural soils can have a significant impact on ecosystem functionality and stability (Yuan et al., 2021; Guo et al., 2022; Tian et al., 2022; Zhou S.-Y.-D. et al., 2023). For example, competing interactions among key taxa can lead to increased diversity of bacterial and fungal communities while minimizing soil organic carbon loss (Gao et al., 2020). Additionally, studies have shown that ecological networks properties can impact how soil microbial communities respond to environmental changes (de Vries et al., 2018; Yuan et al., 2021; Zhou et al., 2021). For example, Yonatan et al. (2022) have suggested that natural microbiomes with weaker interactions tend to be more stable compared to those with stronger interactions. Given the pivotal role of microbial networks for determining the ecosystem functionality and responses of wetland communities to disturbances, a comprehensive understanding of how climate warming influence microbial networks among native plant wetlands and invasive plant wetlands can shed light on the mechanisms and the direction of response of the different coastal wetland ecosystems to climate change in the future.

To investigate whether there are differences in the response of soil microbes (prokaryotes and fungi) to climate warming between native plant wetlands and invasive plant wetlands, we studied the temporal dynamics and networks of soil microbes under experimental warming in two coastal wetlands dominated by native *Phragmites australis* (*P. australis*) and invasive *S. alterniflora*, respectively. Our objectives were to determine: (1) How experimental warming affects soil microbes in coastal wetlands; (2) whether there are variations in the response of soil microbes to warming between native *P. australis* and invasive *S. alterniflora*; (3) how warming-induced changes shape ecosystem stability and functioning.

## 2 Materials and methods

### 2.1 Study area and experimental design

**Figure 1:**
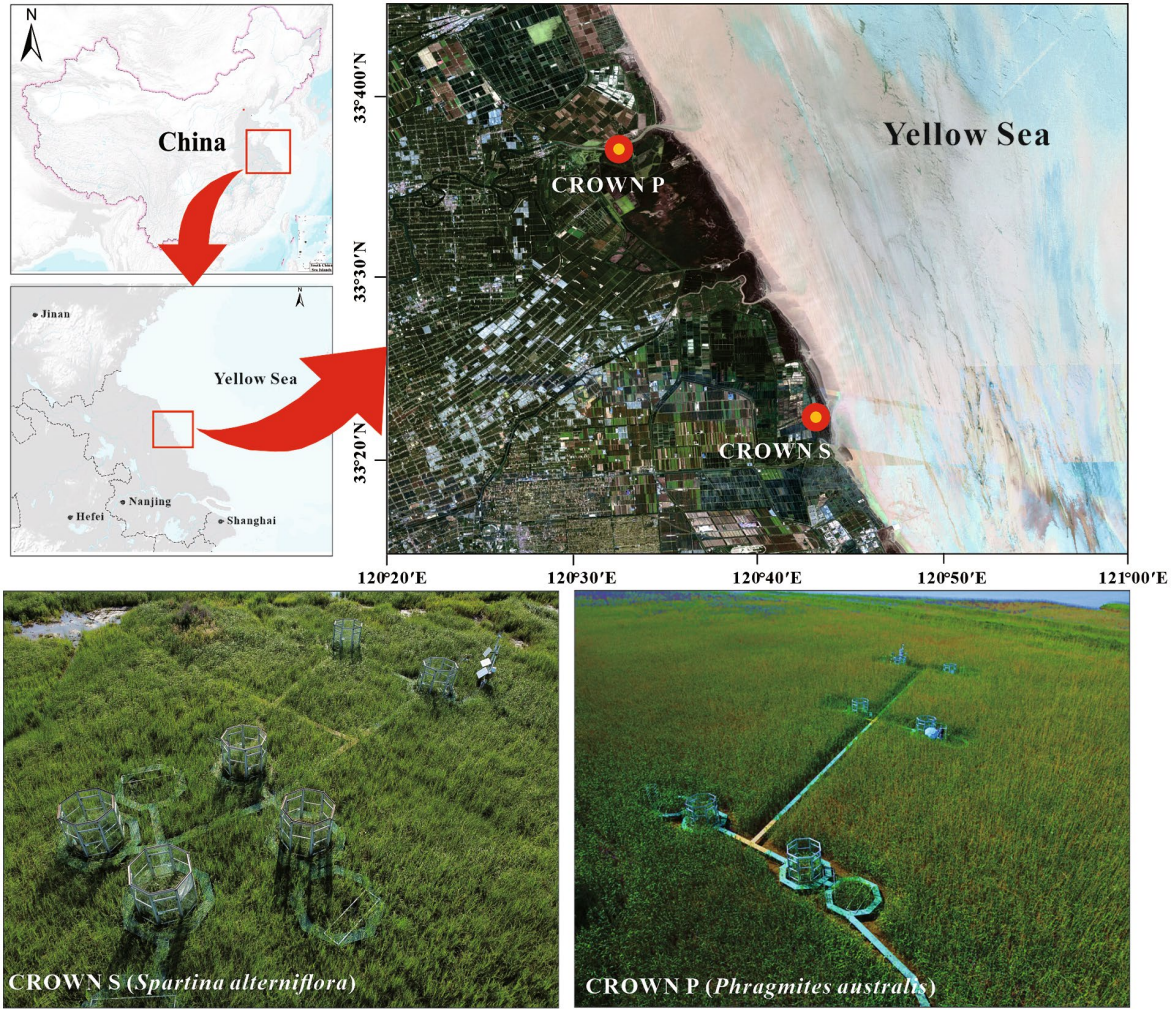
Experimental locations and design of CROWN S site (*Spartina alterniflora*) and CROWN P site (*Phragmites australis*) in the coastal wetland of Yancheng, Jiangsu Province, China.

The field experiment is a part of Coastal-wetland Research On Warming Network (CROWN), located in the coastal wetland of Yancheng, Jiangsu Province, China, with an average annual tidal ranged 3–5 m (Figure 1). As described previously, the experiment was carried out in a temperate-subtropical climate transition zone, experiencing four distinct seasons (Zhou P. et al., 2023). The average air temperature is 15.5°C, and the average annual precipitation is 1,015 mm. The experimental site, CROWN S (33°22′N, 120°43′E) and CROWN P (33°36′N, 120°32′E) was dominated by C_3_ grasses (*S. alterniflora*) and C_4_ grasses (*P. australis*), respectively. Starting from December 2017, the experimental plots were subjected to temperature manipulation to simulate climate warming. Each site consisted of six blocks, with each block containing one control plot and one warming plot. The warming plots were designed using open-top chambers (OTCs), which were octagonal structures measuring 2.7 m in height and covering an area of 5.54 m^2^. As delineated in our prior studies (Yu et al., 2023), these structures can be analogized to diminutive greenhouses. Each of the octagonal facets spans 1.07 meters in width, unified by the interconnection of aluminum alloy frames. The facets are constructed from 4 mm thick stalinite glass, which is clear and transparent, boasting a transmittance rate of 92%. This design facilitates the absorption and retention of incoming solar radiation, thereby enhancing heating without inhibiting photosynthetic processes. Furthermore, by blocking turbulent exchanges and reflecting a portion of the long-wave radiation emitted from the ground back to plants and surface soil, the internal temperature of the chambers is naturally elevated. In the control plots, an equivalent-sized woody structure was installed to minimize environmental disturbances caused by the OTC installation. Monitoring data from 2018 to 2020 showed that the OTCs led to a significant average increase in air temperature of 0.54°C at CROWN P and 0.69°C at CROWN S (Zhou P. et al., 2023). Notably, Due to the passive warming nature of OTCs, the degree of temperature increase cannot be precisely controlled, resulting in variations across different seasons. In this study, the highest temperature increase was observed in the summer, while the lowest was in the winter (Zhou P. et al., 2023).

### 2.2 Field measurements, sampling, and physicochemical analysis

Temperature sensors (HMP155A, Vaisala, Finland) placed at the center of each plot were used to record air temperature every 10 min in the warming/control plots. The pen salinity meter (SA287, HAZFULL, China) was used to measure the salinity of wetland water in the field. Ecosystem carbon exchanges, including net ecosystem exchange (NEE), ecosystem respiration (R_eco_), and gross primary productivity (GPP), were seasonally measured by an Ultra-Portable Greenhouse Gas Analyzer (Los Gatos Research, Quebec, Canada) along with a static chamber.

From May 2018 to January 2020, soil samples were collected seasonally (May 2018, July 2018, September 2018, November 2018, April 2019, July 2019, September 2019, November 2019, and January 2020) from the surface layer (0–10 cm) in three control and three warming plots at each CROWN site. Each sample was a composite of five soil cores (Diameter × Depth = 2.5 cm × 10 cm) obtained using a soil drill. Finally, 108 soil surface samples (9 batches) were collected and stored in a freezer at −80°C until processing. Before microbial and physicochemical analysis, visible roots and stones were manually removed from the composited soil in the lab.

Soil moisture content was determined by calculating the weight loss after freeze drying the samples. The bulk density (BD) of the soil was determined by the ring knife method, where the dry weight of the soil sample in the ring knife was divided by the volume of the ring knife. Soil pH was measured with a pH meter (Mettler-Toledo model GmbH 8,603) in a 1:2.5 sediment-to-water suspension. Total organic carbon (TOC) and total nitrogen (TN) were determined by combusting the dried samples in a CHNS analyzer (Perkin-Elmer model 2,400). Major components SiO_2_, Al_2_O_3_, Fe, K_2_O, CaO, Na_2_O, MgO, and P_2_O_5_ in the soils were measured using X-ray fluorescence spectroscopy (ZSX Primus II, Rigaku, Japan).

### 2.3 DNA extraction, PCR, Illumina sequencing, and quantitative PCR

We extracted microbial DNA from 250 mg of freeze-dried, well-mixed soil for each sample (top 10 cm, bulk soil) using the DNeasy Powersoil Pro Kit (QIAGEN, Germany). The quality and purity of the extracted DNA were assessed using an Ultramicro spectrophotometer (DS-11, DeNovix, USA).

To determine the microbial composition of soil, 16S ribosomal RNA (rRNA) gene and ITS fragment amplicons were prepared and sequenced with the Illumina Miseq platform. Following a previously established protocol, 515FmodF (5′-GTGYCAGCMGCCGCGGTAA-3′) and 806RmodR (5′-GGACTACNVGGGTWTCTAAT-3′) targeting the V4 hypervariable region of prokaryotic (bacterial and archaeal) 16S rRNA genes (Walters et al., 2015), and ITS1F (5′-CTTGGTCATTTAGAGGAAGTAA-3′) and ITS2R (5′-GCTGCGTTCTTCATCGATGC-3′) for fungal ITS fragment (Adams et al., 2013) were used in this study. The amplicons from each sample underwent gel purification and were utilized for the preparation of the sequencing library. Subsequently, the libraries were sequenced using the Illumina Miseq platform with 2 × 250 bp reads. The amplicon sequencing data were processed using the online platform of the Majorbio Cloud Platform.^1^ This involved demultiplexing, trimming, and quality score-based cleaning. The forward and reverse sequences were merged and clustered into operational taxonomic units (OTUs) at 97% similarity for both the 16S rRNA gene and the ITS. Singleton OTUs was eliminated, and to normalize the samples, 28,500 sequences of the 16S rRNA gene and 33,000 sequences of the ITS fragment were randomly resampled for each sample.

Total prokaryotic 16S rRNA gene and fungal ITS fragment abundances were measured for samples from 2019 by quantitative PCR (Q-PCR) using 515FmodF and 806RmodR primers for prokaryotes and ITS1F and ITS2F primers for fungi. All sample were run as technical triplicates on ABI7300 Q-PCR, using the 2X ChamQ SYBR color qPCR Master Mix with 2 uL DNA per 20-ul reaction and primers at a final concentration of 0.4 uM. The PCR program was 3 min at 95°C initially, followed by 40 × (5 s at 95°C, 30 s at 60°C, 60 s at 72°C), and a melt curve (60°C–95°C). Efficiencies were 97.39% for prokaryotes, with an R^2^ of 0.9919, and 101.47% for fungal, with an R^2^ of 0.9997.

### 2.4 Microbial ecological network analysis

We analyzed prokaryotic and fungal co-occurrence networks for each site and treatment separately. Each network consisted of 27 samples from various sampling times. To ensure reliable data associations, we included only OTUs that were present in 21 or more (>75%) samples for analysis. All networks were constructed using the Molecular Ecological Network Analysis pipeline (MENAP^2^) and were based on Spearman correlations (Deng et al., 2012). A threshold of r ≥ 0.820 and *p* < 0.001 were determined for network construction using an approach based on random matrix theory (Zhou et al., 2010; Yuan et al., 2021). Visualization of the networks was performed using Gephi software (0.10.1). The roles of nodes in the network were identified by calculating within-module connectivity (Zi) and among-module connectivity (Pi) (Guimerà and Nunes Amaral, 2005). The criteria used for categorizing nodes into module hubs, connectors, network hubs, and peripherals were based on previous studies (Zhou et al., 2011; Shi et al., 2016; Yuan et al., 2021). To assess the robustness of the microbial network, we calculated the proportion of the remaining species in the network after the removal of random or targeted nodes. In this study, we calculated the robustness when 50% of random nodes or keystone nodes were removed, as previously described (Yuan et al., 2021; Guo et al., 2022).

### 2.5 Statistical analysis

The impact of warming and plant invasion on soil variables and ecosystem carbon fluxes were assessed through one-way analysis of variance (ANOVA) in SPSS Statistics 27.0. The combined impacts of research site, sampling time, and experimental warming on gene abundance and alpha diversity indices were analyzed using linear mixed-effects models (Guo et al., 2022; Wu et al., 2022). The effects on the microbial community were tested using Adonis based on the Bray-Curtis distance. Additionally, we utilized Principal coordinate analysis (PCoA) to visually represent the patterns of the microbial community across different sampling times, coupled with ANOSIM for inter-group difference testing. Time-decay relationships (TDRs) were calculated as the slopes of ordinary least-squares regression lines for the relationships between the time interval and community similarities (Bray–Curtis metric). We conducted Adonis/ANOSIM to test for differences in the entire microbial community composition (Anderson, 2001). This analysis considered the factors of experimental warming, sampling time. To explore the associations between microbial community composition and soil variables, as well as ecosystem carbon fluxes, we employed Mantel-tests. All statistical analyses were performed using R version 4.0.5, along with specific packages such as vegan, picante, nlme, and lmerTest, unless otherwise stated. Finally, we utilized the FAPROTAX and Funguild databases for functional annotations of prokaryotes and fungal taxa, respectively.

## 3 Results

### 3.1 Soil variables and ecosystem carbon flux (NEE, R_eco_, and GPP)

In coastal wetlands, the majority of soil variables remain relatively constant under both experimental warming and varying time conditions (Table 1; Supplementary Table S1; Supplementary Figure S1). There were no significant differences in soil variables between the warming and control treatments, except for a significant increase in salinity from 15.2 ± 0.7 to 17.1 ± 0.6 ppt in the warming treatment at CROWN S (*p* = 0.041, Table 1). Despite the levels of NEE, R_eco_, and GPP appeared to decrease in the warming versus control sites, the warming effects of all those metrics were not statistically supported (*p* > 0.05). In Table 1, no matter in the warming or control treatments, CROWN P exhibited lower NEE and R_eco_ values compared to CROWN S. Combined with significantly higher TOC at CROWN P, the carbon uptake capacity of CROWN P was greater than that of CROWN S. Over the period 2018–2020, aside from the pronounced seasonal fluctuations of ecosystem carbon fluxes (NEE, R_eco_, and GPP), TOC, TN, and Moisture (*p* = 0.000–0.0350), metrics including soil pH, salinity, SiO_2_, Al_2_O_3_, K_2_O, Fe_2_O_3_, and P_2_O_5_ remained constant in the coastal wetlands, with marginally significant or insignificant seasonality (*p* = 0.082–0.804, Supplementary Figure S1; Supplementary Table S1). However, at the CROWN P site, there was an abrupt accumulation of sediment that started in April 2019, and the soil pH, TOC, and TN decreased significantly compared to 2018, while the soil moisture increased substantially (Supplementary Figure S1). The levels of TOC, TN, and water content were higher and the pH, salinity, and BD were lower at the CROWN P site compared to the CROWN S site (Table 1).

**Table 1:** The major soil variables and ecosystem carbon fluxes (mean ± SE) in study area.

Soil variables	cCROWN S	wCROWN S	cCROWN P	wCROWN P
pH	8.181 ± 0.049 A	8.173 ± 0.051 A	7.780 ± 0.101 B	7.777 ± 0.104 B
Salinity (ppt)	15.156 ± 0.661 A	17.056 ± 0.622 B	11.433 ± 0.750 C	10.622 ± 0.887 C
Moisture (%)	42.947 ± 1.910 A	41.591 ± 1.848 A	74.757 ± 3.987 B	75.993 ± 3.383 B
BD (g cm^−3^)	1.231 ± 0.037 A	1.280 ± 0.035 A	0.881 ± 0.042 B	0.858 ± 0.037 B
TN (mg g^−1^)	0.986 ± 0.064 A	0.962 ± 0.057 A	2.628 ± 0.131 B	2.907 ± 0.142 B
TOC (mg g^−1^)	5.275 ± 0.214 A	5.411 ± 0.179 A	14.904 ± 0.392 B	16.275 ± 0.587 B
NEE (g m^−2^ h^−1^)	−1.161 ± 0.185 A	−0.927 ± 0.156 A	−1.472 ± 0.246A	−1.16 ± 0.238A
Reco (g m^−2^ h^−1^)	1.023 ± 0.073 A	0.962 ± 0.075 A	0.884 ± 0.144 A	0.823 ± 0.136 A
GPP (g m^−2^ h^−1^)	2.1845 ± 0.232 A	1.889 ± 0.194 A	2.356 ± 0.323 A	1.984 ± 0.316 A

Differences between sites and treatments were tested by analysis of variance. Different capital letters indicate significant differences between treatment and sites at *p* < 0.05. CROWN S, *Spartina alterniflora* wetland; CROWN P, *Phragmites australis* wetland; cCROWN S, control treatment at CROWN S; wCROWN S, warming treatment at CROWN S; cCROWN P, control treatment at CROWN P; wCROWN P, warming treatment at CROWN P; BD, bulk density; TN, total nitrogen; TOC, total organic carbon; GPP, gross primary productivity; R_eco_, ecosystem respiration; NEE, net ecosystem exchange.

### 3.2 Microbial abundance, diversity, and composition,

We investigated the influence of warming and wetland type (native *Phragmites australis* wetland vs. invasive *Spartina alterniflora* wetland) on soil prokaryotic and fungal communities at CROWN sites over nine consecutive seasons from 2018 to 2020. Our findings showed that the wetland type had the most significant influence on the abundance, diversity, and composition of soil microorganisms, followed by sampling time (Table 2). The effect of warming on the abundance, diversity, and composition of soil microorganisms was found to be insignificant (*p* > 0.083, Table 2; Supplementary Figure S2).

**Table 2:** The combined impacts of wetland type (native *Phragmites australis* wetland vs. invasive *Spartina alterniflora* wetland), sampling time, and experimental warming on soil microbes.

	Prokaryotes						Fungi					
	Abundance		Shannon index		Community		Abundance		Shannon index		Community	
	F	*p*	F	*p*	F	*p*	F	*p*	F	*p*	F	*p*
Type	42.608	**0.000**	23.103	**0.000**	120.662	**0.001**	20.789	**0.001**	21.385	**0.000**	80.478	**0.001**
Time	0.465	0.495	30.385	**0.000**	6.748	**0.002**	0.004	0.950	2.473	0.116	6.003	**0.001**
Warming	0.402	0.526	0.375	0.541	1.341	0.208	0.369	0.543	0.044	0.834	1.882	0.083
Type × Time	0.563	0.453	28.360	**0.000**	8.506	**0.001**	0.007	0.933	2.865	0.091	6.454	**0.001**
Type × Warming	0.321	0.571	0.059	0.808	1.192	0.265	0.041	0.839	0.100	0.752	1.654	0.118
Time × Warming	1.974	0.160	0.216	0.642	0.759	0.543	0.065	0.799	0.155	0.694	0.873	0.459
Type × Time × Warming	1.668	0.197	3.719	0.054	0.777	0.460	0.170	0.681	0.007	0.934	0.837	0.493

The differences between groups of microbial abundance and Shannon were tested by linear mixed-effects models, and the microbial community were tested by Adonis based on the Bray-Curtis distance. Statistical significance (*p* < 0.05) is indicated in bold.

**Figure 2:**
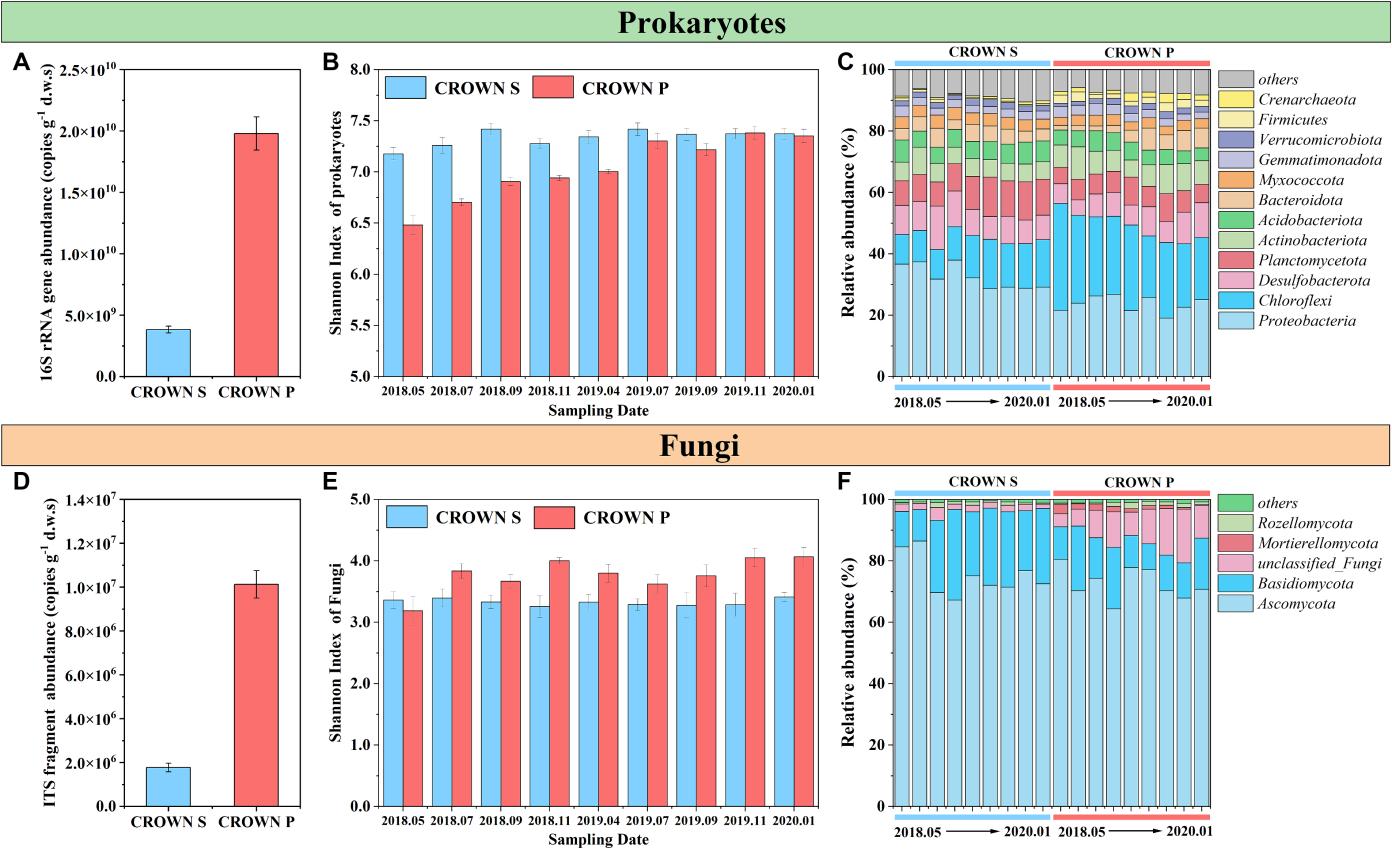
Microbial abundance, diversity, and composition at CROWN S and CROWN P site. **(A)** The absolute abundance of the 16S rRNA gene. **(B)** The Shannon index of prokaryote diversity at the OTU level across all sampling dates. **(C)** Prokaryotic taxonomic composition at the phylum level across all sampling dates. **(D)** The absolute abundance of the ITS fragment. **(E)** The Shannon index of fungal diversity at the OTU level across all sampling dates. **(F)** Taxonomic composition of fungi at the phylum level across all sampling dates. Error bars represent standard errors. To increase the statistical power, this analysis no longer differentiates between the group subjected to warming and the control treatment. CROWN S, *Spartina alterniflora* wetland; CROWN P, *Phragmites australis* wetland.

The absolute abundances of the 16S rRNA gene and ITS fragment in the wetland soil remained relatively constant over time and under the warming treatment (Table 2; Supplementary Figure S3). However, a markedly higher abundance of the 16S rRNA gene and ITS fragment was observed at CROWN P compared to CROWN S (Figures 2A,D). Furthermore, the results of Pearson analysis showed that the absolute abundance of these genes were strongly correlated with the levels of TOC, P_2_O_5_ and TN (*r* = 0.659–0.853, *p* < 0.001, Supplementary Table S2). For alpha diversity, the richness and diversity of prokaryotic microorganisms were significantly higher at CROWN S, while the opposite trend was observed for fungi (Supplementary Table S3). Experimental warming did not have a significant impact on the alpha diversity of both prokaryotes and fungi (Table 2; Supplementary Table S3), indicating their resilience to warming. Moreover, the diversity (e.g., Shannon index) of prokaryotes exhibited temporal shifts (Table 2), with a significant increasing trend over time at the CROWN P site (Figure 2B). This trend was strongly correlated with salinity (*r* = 0.685, *p* < 0.001). Conversely, diversity at the CROWN S site remained relatively constant throughout the study period (Figures 2B,E; Supplementary Table S4).

From Figures 2C,F, the dominant phyla in the prokaryotic microbial community were *Proteobacteria*, *Chloroflexi*, *Desulfobacterota*, *Planctomycetota*, and *Actinobacteriota*, while the fungal community was mainly composed of *Ascomycota* and *Basidiomycota*. At the class level, *AlphaProteobacteria* and *GammaProteobacteria* contributed to nearly all phylum *Proteobacteria*, while the most abundant class in the phylum *Chloroflexi* was *Anaerolineae*. The microbial community composition of both prokaryotes and fungi showed marked differences between CROWN S and CROWN P (Table 2). Specifically, for prokaryotes, *Proteobacteria*, *Desulfobacterota*, and *Planctomycetota*, *Acidobacteriota* were more abundant in the soil at CROWN S compared to CROWN P (*p* < 0.007), whereas *Chloroflexi*, *Actinobacteriota*, and *Firmicutes* were more abundant at CROWN P than at CROWN S (*p* < 0.001). As for fungi, *Basidiomycota* was notably more abundant at CROWN S than at CROWN P (*p* < 0.001). However, *Mortierellomycota* were significantly more abundant at CROWN P than at CROWN S (*p* < 0.05). The correlation analysis indicated that the phyla, which differ significantly between CROWN S and CROWN P, were highly related with the variation of soil properties (Supplementary Table S5). The relative abundances of *Proteobacteria*, *Planctomycetota*, and *Acidobacteriota* were negatively correlated with the levels of TOC, TN, P_2_O_5_, and other nutrient elements. On the other hand, *Chloroflexi*, *Actinobacteriota* and *Firmicutes* showed an opposite trend, with their relative abundances positively correlated with the levels of TOC, TN, P_2_O_5_, and other nutrient elements. *Desulfobacterota* only showed a significant positive correlation with salinity and Na_2_O. As for fungi, *Mortierellomycota* showed significant correlations with various soil properties, while *Basidiomycota* showed relatively weaker correlations with soil parameters.

Statistical tests (ANOSIM and Adonis) using Bray-Curtis, weighted Unifrac, and unweighted Unifrac distance metrics (Supplementary Table S6) revealed that the community composition of both prokaryotes and fungi remained constant in the experimental warming treatments. However, there was an exception to this constant. The fungal community at CROWN P was significantly different in the warming treatment when the analysis was performed with Bray-Curtis distance metrics. That difference accounted for 4.3% of the total variance (Supplementary Table S6).

**Figure 3:**
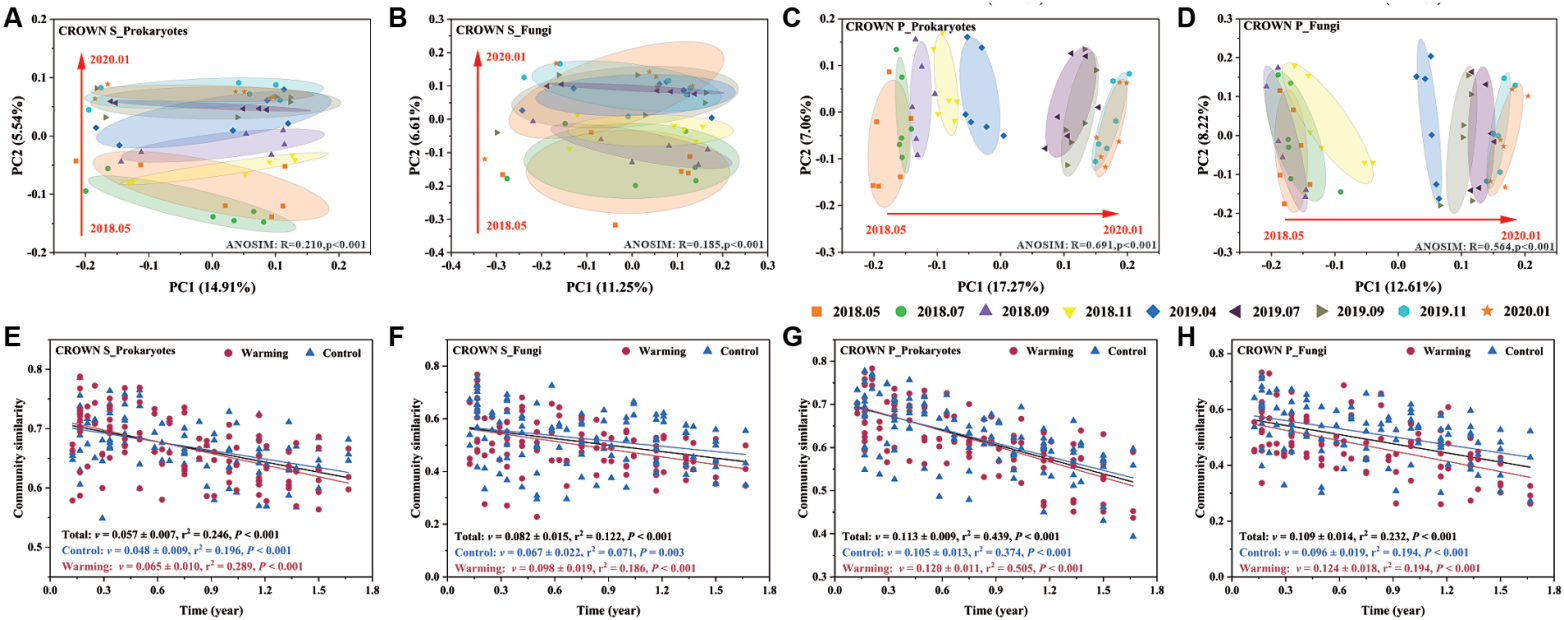
The temporal evolution of soil microbial communities at the CROWN S and CROWN P sites. **(A–D)** Principal coordinate analysis of the temporal changes in microbial communities at CROWN S and CROWN P. The analysis was performed based on unweighted UniFrac distance metrics. The information for other distance metrics (Bray-curtis and weighted UniFrac) is shown in Supplementary Figures S4, S5. To increase the statistical power, this analysis no longer differentiates between the group subjected to warming and the control treatment. **(E–H)** The time-decay relationships (TDRs) of soil prokaryotic and fungal communities under warming and control conditions. Community similarity was calculated as Bray-Curtis distance metrics. The TDR value (*v*) represents the absolute slope of the linear regression and indicates the rate of temporal turnover of the soil microbes. CROWN S, *Spartina alterniflora* wetland; CROWN P, *Phragmites australis* wetland.

Experimental warming had no significant effect on the structure of the soil microbial communities. However, the timing of sampling did have a significant impact (Table 2). Sampling time explained 20–36% of the variance in the prokaryotic microbial community and 20–33% of the variance in the fungal community (Supplementary Table S7). It is important to note that the microbial community composition in the study area has undergone significant changes over time, but it has not exhibited seasonal cyclic patterns (Figures 3A–D; Supplementary Figures S4, S5). To better understand the temporal changes in microbial community composition, we analyzed the time-decay relationships (TDRs) of the soil prokaryotes and fungi using linear regressions between community similarity and temporal distance. The slope of the linear regression, represented by the TDRs value (*v*), indicates the turnover rate of soil microbes over time. Our results showed that the *v* value of the microbial community was significantly higher at CROWN P site compared to the CROWN S site, and the *v* value was significantly higher for the fungi compared to the prokaryotes (Figures 3E–H). Furthermore, the *v* values for prokaryotes and fungi at the CROWN S and CROWN P sites were considerably higher in the warming treatments (Figures 3E–H).

### 3.3 Microbial community co-occurrence network

**Figure 4:**
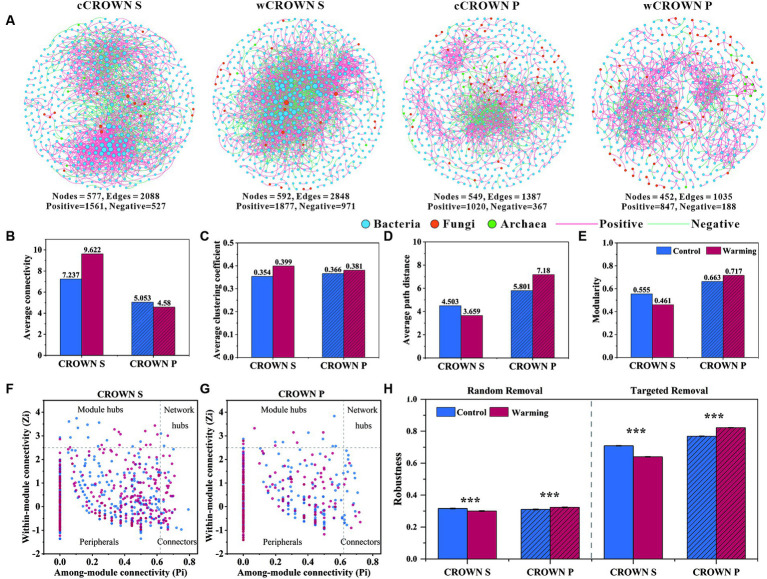
The co-occurrence networks of microbial communities in warming and control treatments at CROWN S and CROWN P sites. **(A)** Overall dynamics of microbial co-occurrence networks under warming and control. The nodes represent unique OTUs in the datasets. The size of each node is proportional to the connectivity. **(B–E)** Topological properties of soil microbial co-occurrence networks in the warming and control treatments. **(F,G)** Zi-Pi plots to identify putative keystone taxa under warming (red) and control (blue) treatments. **(H)** Stability of microbial networks measured by robustness. In the case of random removal, robustness was equated to the proportion of taxa that remained when 50% of the taxa were randomly removed from each of the microbial networks. In the case of targeted removal, robustness was equated to the proportion of taxa that remained when 50% of the keystone nodes (module hubs, connectors, and network hubs) were removed from each of the microbial networks. CROWN S, *Spartina alterniflora* wetland; CROWN P, *Phragmites australis* wetland; cCROWN S, control treatment at CROWN S; wCROWN S, warming treatment at CROWN S; cCROWN P, control treatment at CROWN P; wCROWN P, warming treatment at CROWN P.

Using the abundance of prokaryotic and fungal OTUs from each site, we built twenty-two co-occurrence networks to explore the associations of microorganisms in different sites, treatments, and sampling time (Figure 4A; Supplementary Figure S6). The co-occurrence networks exhibited scale-free (R^2^, 0.927–0.993) and small-word characteristics (the average path distance, 1–7.180), indicating that they were not formed by random chance. As illustrated in Figure 4A and Supplementary Figure S6, bacterial dominance was observed in the microbial network of the coastal wetland soil, with fungi and archaea having lower representation. In addition, the CROWN S site had a larger and more complex network compared to the CROWN P site (Figure 4). Moreover, the effects of warming treatments on the topological properties of soil microbial co-occurrence networks differed significantly between CROWN S and CROWN P sites (Figures 4A–E; Supplementary Table S8), with warming enhanced complexity and connectivity in CROWN S and diminished them in CROWN P. On the temporal scale, the complexity of microbial network showed a significantly increasing trend over time (*p* < 0.026), while other topological properties (e.g., Average connectivity and Modularity) remained constant (Supplementary Figure S6).

The altered complexity of a network could lead to changes in the role of individual members within the network. In this study, we identified a total of 24 (16) module hubs, 53 (25) connectors, and 3 (0) network hubs in the CROWN S (CROWN P) microbial network. These nodes can be identified as keystone nodes that was essential in shaping network structure (Figures 4F–G; Supplementary Table S9). The CROWN S site had the highest number of module hubs, connectors, and network hubs. In addition, warming caused the number of keystone nodes to increase (decrease) at the CROWN S (CROWN P) site (Figures 4F–G; Supplementary Table S9). Most of the key taxa in both the CROWN S and CROWN P networks belonged to abundant phyla: 25.4% were *Proteobacteria*; 21.1% were *Chloroflexi*; and 17.5% were *Planctomycetota* (Supplementary Table S9). Some OTUs were found in multiple networks, serving as module hubs, connectors, or network hubs (Supplementary Table S9). For instance, OTU720, which belonged to the genus *Halioglobus* in the *Proteobacteria*, was a module hub in both the cCROWN S and wCROWN S networks. However, it is noteworthy that only 3.4% of all the keystone nodes were common between the warming and control treatments (Supplementary Table S9), indicating that warming induced alterations in the network structure at the level of keystone node.

All the microbial community co-occurrence networks that we detected were scale-free, modular, and small-world. We hypothesized that this fact might have significant implications for microbially mediated ecosystem stability. To test this hypothesis, we simulated species extinctions and calculated the robustness of the microbial community co-occurrence networks. When species were randomly lost or keystone nodes (module hubs, connectors, and network hubs) were targeted removed, the networks of CROWN P showed significantly greater robustness in the warming treatment compared to the control treatment (Figure 4H). Conversely, the networks of CROWN S showed significantly lower robustness in the warming treatment compared to the control treatment (Figure 4H). These results suggested that under experimental warming, the microbial community co-occurrence network become more stable in CROWN P but less stable in CROWN S.

### 3.4 Discerning drivers and functions of the soil microbial community

**Figure 5:**
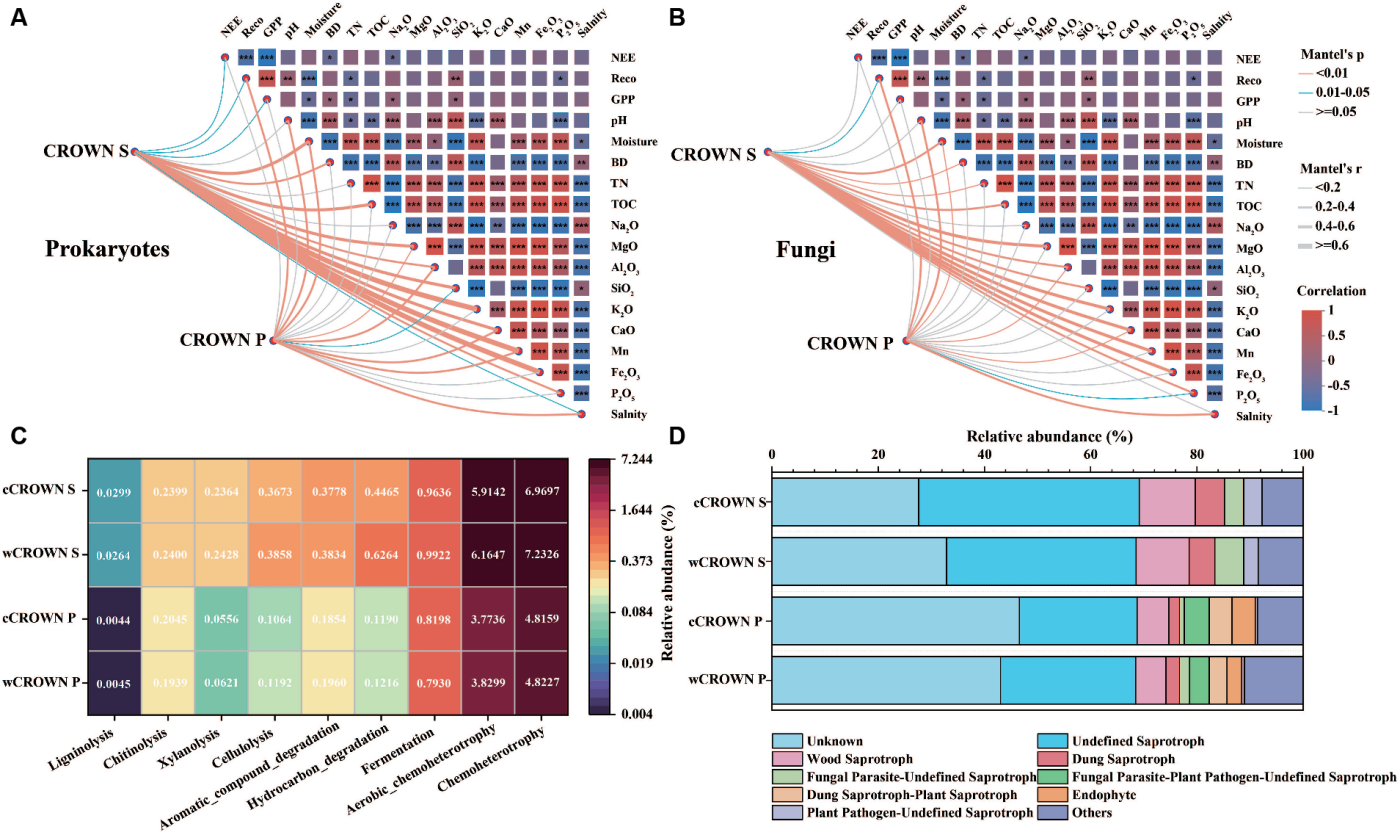
Relationships between the microbial community, soil variables, ecosystem processes, and community functional traits. **(A)** Correlations of the prokaryotic community composition (Bray-Curtis distance) with soil variables and ecosystem carbon fluxes. **(B)** Correlations of the fungal community composition (Bray-Curtis distance) with soil variables and ecosystem carbon fluxes. **(C)** Functional prediction for prokaryotes with the Faprotax database. **(D)** Functional prediction for fungi with the FUNGuild database. CROWN S, *Spartina alterniflora* wetland; CROWN P, *Phragmites australis* wetland; cCROWN S, control treatment at CROWN S; wCROWN S, warming treatment at CROWN S; cCROWN P, control treatment at CROWN P; wCROWN P, warming treatment at CROWN P. NEE, net ecosystem exchange; R_eco_, ecosystem respiration; GPP, gross primary productivity; BD, bulk density; TN, total nitrogen; TOC, total organic carbon.

Differences in soil microbial communities were observed between CROWN S and CROWN P sites (Table 2). Our analysis, using Mantel test, revealed that the composition of the soil microbial communities was mainly correlated with TOC, followed by Na_2_O, K_2_O, P_2_O_5_, and TN (Supplementary Table S10). Additionally, we found that the soil microbial communities at CROWN S showed generally stronger correlations with various environmental parameters compared to CROWN P (Figures 5A,B). Specifically, at CROWN S, the microbial community composition was significantly influenced by the major components of the soil (K_2_O, CaO, MgO, Mn, Fe_2_O_3_), moisture, and TOC (Figure 5A). At CROWN P, the microbial community composition was significantly shaped by CaO, salinity, and pH (Figure 5B). Notably, these correlations were generally robust in both the warming and the control treatment, although the correlations in some cases were relatively weak (Supplementary Figure S7). Furthermore, at both the CROWN S and CROWN P sites, there was a close association between the composition of the soil microbial community and R_eco_ (Figures 5A,B).

To evaluate the differences in carbon degradation potential between sites and treatments, we used the Faprotax and FunGuild databases to predict the functions of the prokaryotes and fungi, respectively, with OTUs. The results showed that the proportion of prokaryotic OTUs involved in carbon degradation were significantly higher at CROWN S than at CROWN P (*p* < 0.05, Figure 5C; Supplementary Table S11), and the warming treatments generally resulted in an increased proportion of OTUs involved in carbon degradation (Figure 5C). Based on their nutritional modes, eight guilds of fungi (abundances >2%) were identified from the three major fungal categories: saprotrophs, symbiotrophs, and pathotrophs (Figure 5D). Among these categories, the relative abundances of undefined saprotrophs (22.2–41.5%) and wood saprotrophs (5.7–10.6%) were the highest. A comparison of the two sites revealed that the relative abundance of saprotrophs was significantly higher at the CROWN S site (*p* < 0.05, Supplementary Table S12), whereas the proportion of symbiotrophs was significantly higher at the CROWN P site (*p* < 0.01, Supplementary Table S12). There was no discernible evidence of significant differences in the relative abundances of the various guilds between the control and warming treatments at either site (Supplementary Table S12).

## 4 Discussion

### 4.1 Distinct soil microorganisms across invasive *Spartina alterniflora* and native *Phragmites australis*

In the present study, prokaryotic and fungal communities were studied in quantitative and qualitative terms over the coastal wetland. Compared to the CROWN P site, the CROWN S site exhibited higher microbial abundance (Figures 2A,D). This can be attributed to the higher levels of nutrients (such as TOC, TP, TN, etc.) in the soil at the CROWN P site (Supplementary Table S2). Organic carbon acts as the primary carbon source for microbial growth and reproduction, while phosphorus and total nitrogen are essential nutrients for microbial growth (Stotzky and Norman, 1961; Li et al., 2015). The favorable conditions of high TOC, P_2_O_5_, TN create a conducive environment for microbial growth and reproduction (Yang et al., 2019a,b). It is worth emphasizing that in this study, we found minimal differences between seasons in the abundances of soil microorganisms in coastal wetlands (Supplementary Figure S3). Whether in the hot summer (July) or cold winter (November), the fact that they were able to maintain a relatively constant abundance indicated that these microbial communities were able to adapt to the environmental changes associated with the different seasons while retaining their ecological functions. This relatively constant abundance may also be attributed to the homogeneity of soil properties in coastal wetlands, which may in part reflect the influence of robust hydrological fluctuations like tidal cycles, which mitigate the effects of soil heterogeneity.

The diversity of prokaryotic microbes at CROWN P gradually increased over time and was closely associated with changes in salinity (*R* = 0.685, *p* < 0.001) (Figure 2B). Interestingly, when the soil salinity at CROWN P approached that of CROWN S, the diversity index at CROWN P also approached that of CROWN S. These findings indicated that salinity in the study area played a crucial role in shaping the prokaryotic microbial diversity. In the soil, salinity can alter the distribution of roots, enhance soil heterogeneity, and consequently lead to higher diversity and richness of soil microorganisms (Yang et al., 2016). In Figure 2E and Supplementary Table S3, the richness and diversity of fungi were significantly higher at CROWN P than that at CROWN S. The difference in fungal richness and diversity could be due to the stronger symbiotic relationship between *P. australis* and soil fungi, whereas in comparison, the symbiotic relationship between *S. alterniflora* and soil fungi is less common or harmful to fungal survival (Figure 5D; Supplementary Table S12), leading to reduced fungal richness and diversity. This discovery was consistent with the results of earlier research, which have suggested that *S. alterniflora* exhibits lower rates of fungal colonization than *P. australis* (Liang et al., 2016).

Based on microbial taxonomic information, most of the prokaryotic and fungal groups observed in other coastal areas (An et al., 2019; Yang et al., 2019b; Lin et al., 2022; Hussain et al., 2023) were also identified in this study. Nevertheless, noticeable differences were evident in the composition of the prokaryotic and fungal communities between CROWN S and CROWN P soils. Among them, *AlphaProteobacteria*, *Planctomycetota*, and *Acidobacteriota* are typical oligotrophic bacteria, capable of adapting well to low-nutrient environments (Cui et al., 2019; Zhang et al., 2019; Wang et al., 2021a), whereas *Actinobacteriota* and *Firmicutes* are classified as copiotrophic bacteria, growing rapidly in nutrient-rich environments (Li et al., 2021; Yang et al., 2023). This corresponds well with our Pearson analysis results (Supplementary Table S5), and effectively explaining the differences in their relative abundance between CROWN S (low-nutrient environments) and CROWN P (high-nutrient environments). Surprisingly, *Chloroflexi*, typically oligotrophic bacteria (Yang et al., 2023), were more abundant in the nutrient-rich environment of CROWN P compared to CROWN S, and their abundance was positively correlated with levels of TOC, TN, and other nutrients (Supplementary Table S5). This may be due to hydrological factors, as CROWN P soil has high soil moisture content (Table 1), and subsequence anaerobic conditions favor the growth of *Anaerolineae* (the most abundant class in *Chloroflexi*) (Yamada and Sekiguchi, 2009). Pearson correlation analysis also showed a significant positive correlation between the relative abundance of *Chloroflexi* and soil moisture content (Supplementary Table S5). *Desulfobacterota*, dominant among sulfate-reducing bacteria communities in salt marshes and known to increase in abundance after invasion by *S. alterniflora* (Zeleke et al., 2013; Zhang et al., 2020), was consistent with our study. Pearson correlation analysis indicated that its relative abundance was significantly and positively correlated only with salinity and Na_2_O (Supplementary Table S5), suggesting that salinity primarily controls its distribution. Regarding fungi, Supplementary Table S5 shows that *Mortierellomycota* is highly correlated with various soil parameters, while *Basidiomycota* shows weaker correlations. Therefore, the greater relative abundance of *Basidiomycota* at CROWN S compared to CROWN P may be mainly attributed to plant factors, as previous research has indicated that *S. alterniflora* is rich in recalcitrant carbon compounds (such as lignin and cellulose) (Schneider et al., 2012), and *Basidiomycota* plays a pivotal role in breaking down these compounds (Yang et al., 2009).

### 4.2 Changes in soil microbial communities over time

As shown in Table 2 and Supplementary Table S7, the soil microbial communities in the coastal wetlands changed significantly over time. However, unlike grass and forest ecosystems (Waldrop and Firestone, 2006; Fu et al., 2020; Luo et al., 2020; Zhou S.-Y.-D. et al., 2023), the composition of soil microbial communities in the coastal wetlands did not exhibit a seasonal cyclic pattern over time (Figures 3A–D; Supplementary Figures S4, S5). Generally, the seasonal periodicity of soil microbial communities is attributed mainly to environmental factors that undergo seasonal changes, such as soil moisture and type of vegetation (Brockett et al., 2012; Thoms and Gleixner, 2013; Zhou S.-Y.-D. et al., 2023). Within coastal wetland ecosystems, however, vegetation is uniform, and soil homogenization due to semi-diurnal tides is prevalent. We thus inferred that the relatively stable environment of the coastal wetland soils may have been one of the main factors contributing to the absence of seasonal periodicity in the microbial communities. Additionally, based on TDR analysis (Figures 3E–H) and three microbial communities time-dissimilarity patterns which proposed by Gao et al. (2024), we found the microbial communities time-dissimilarity dynamics in study area was linear pattern. This implied in coastal wetlands, the succession of community composition is driven by factors such as continuous selection, constant dispersal rate, and fixed speciation probability, etc. (Sprockett et al., 2020; Custer et al., 2022). Moreover, the TDR results revealed that replacement of native *P. australis* by invasive *S. alterniflora* slowed the rate of microbial succession (Figures 3E–H). This phenomenon may be attributed to the uniform soil properties observed in the CROWN S (Supplementary Figure S1), which do not possess the necessary characteristics to drive microbial succession. On the other hand, the decrease in spatial heterogeneity within soil microbial communities caused by plant invasion led to a lower rate of succession (Li et al., 2022; Zhang et al., 2023). Lastly, our co-occurrence network analysis of soil microbes showed a linear increase in network complexity over time, while connectivity and modularity remained consistent (Supplementary Figure S6). This outcome suggests that the interrelationships within the microbial community in coastal wetland soils grow more intricate as time advances, yet the overall network structure maintains relative stability. Specifically, the linear escalation in network complexity over time signifies the gradual broadening and intensification of interrelationships within the microbial community. This could be attributed to the accumulation and enhancement of ecological processes and interactions within the microbial community at varying time points. Despite the rise in complexity, the network’s connectivity and modularity remain unvarying, denoting the relative stability of the microbial community’s internal structure. This could indicate that while microbial interactions continue to augment, the fundamental patterns and organizational methods of the overall network structure remain largely unaltered.

### 4.3 Warming shapes coastal wetland soil microbial communities

Our analyses found that short-term warming did not have a significant impact on the abundance, diversity, and composition of soil microbial communities in coastal wetlands (Table 2). However, it did accelerate the turnover rates of soil prokaryotes and fungal communities (Figure 3). This result was consistent with a recent study conducted in a grassland ecosystem (Guo et al., 2018). According to the metabolic theory of ecology, temperature plays a key role in determining metabolic rates, which in turn affect various biological processes, including species turnover (Brown et al., 2004; Ren et al., 2017). Specifically, higher temperatures enhance enzyme activity, leading to faster metabolic rates, cell division, nutrient uptake, and metabolite production. This enables microorganisms to reproduce and grow more rapidly in high-temperature environments, thereby promoting faster succession of microbial communities. It is important to note that warming appeared to increase the rates of species turnover, potentially leading to greater spatiotemporal variations in the soil microbial community (Guo et al., 2018, 2019, 2022). As reported in a montane community and a coastal ecosystem coastal, warming resulted in divergent community succession and accelerated temporal scaling (Hillebrand et al., 2010; Gibson-Reinemer et al., 2015). Therefore, the effects of warming on microbial communities may be progressive (DeAngelis et al., 2015), and as warming persists, its influence may become more pronounced over time. The implication is that future climate change scenarios may lead to microbial communities deviate significantly from their current states. There is hence a possibility that these communities will diverge toward multiple alternative states, as noted by Guo et al. (2018). In addition, the significantly larger increased slope of the TDR for fungi than prokaryotes in the warming treatments suggested that fungi were more sensitive to temperature than prokaryotes, which is consistent with previous studies (Guo et al., 2018; Feng et al., 2022; Zhou S.-Y.-D. et al., 2023). This heightened sensitivity of fungi may be attributed to their greater susceptibility to alterations in surface vegetation (Dassen et al., 2017), as supported by our previous research demonstrating the substantial impact of short-term warming on surface vegetation phenological traits (Zhou et al., 2023a).

The analysis of co-occurrence networks of soil prokaryotic and fungal communities revealed that with warming, the topologic parameters of the networks became more (less) complex and connective at CROWN S (CROWN P) (Figures 4A–E). Furthermore, the microbial community co-occurrence network appeared more (less) stable in CROWN P (CROWN S) under experimental warming (Figure 4H). Collectively, these findings showed that as network complexity and connectivity increase, the stability of microbial ecosystems decreases. This phenomenon can be explained by May’s stability theory (May, 2019). In other words, higher complexity and connectivity in highly diverse ecosystems destabilize ecological systems (May, 2019; Yonatan et al., 2022). Similarly, research by de Vries et al. (2018) has revealed that microbial co-occurrence networks characterized by high connectivity and low modularity are associated with low stability. The implication is that warming may increase the resilience of *P. australis* wetland soil to environmental perturbations and lead to greater stability of microbial communities and their functionality (Thébault and Fontaine, 2010; Coyte et al., 2015). Conversely, under future climate change scenarios, *S. alterniflora* wetlands may become more vulnerable to perturbations. Based on robustness analysis (Figure 4H) and May’s stability theory (May, 2019; Yonatan et al., 2022), we inferred that the soil microbial communities in the *P. australis* wetland were more stable compared to those in the *S. alterniflora* wetland. Compared with the microbial network at ambient temperature, experimental warming increased (decreased) the percentage of negative links between species at CROWN S (CROWN P) (Supplementary Table S8). If these associations indicate biological interactions, the negative associations could be due to competition for limited resources (Fuhrman, 2009; Berry and Widder, 2014). This conclusion aligned precisely with the relatively nutrient-poor environment in the CROWN S soil, where experimental warming accelerated the metabolism and succession of soil microbes and thereby intensified interspecies competition for resources (Faust and Raes, 2012; Berry and Widder, 2014). It should be noted that predicting the status of key species in microbial networks is challenging due to their dynamic nature. Nonetheless, our findings indicated that the keystone nodes within these networks differed between the warming and control conditions. These results suggested that experimental warming had the potential to induce structural changes at the level of the keystone node, as documented by Yuan et al. (2021). Of the 121 keystone nodes we identified (Supplementary Table S9), 82 had a relative abundance of less than 0.1% and were classified as rare. This observation underscores the potential significance of species rarity within ecological networks, as previously highlighted (Fahey et al., 2020; Zhou S.-Y.-D. et al., 2023).

### 4.4 Connections between microbial communities, soil properties, and ecosystem function

The composition of the soil microbial community showed a stronger correlation with environmental factors in CROWN S compared to CROWN P (Figure 5). This difference was mainly attributable to the lower levels of soil nutrients in CROWN S. In low-nutrient environments, microorganisms need to intricately regulate their metabolism and resource acquisition to adapt to the constraints of limited nutrient availability (Dai et al., 2022). Consequently, the composition and activity of microbial communities become more vulnerable to environmental factors, including soil moisture and concentrations of nitrogen, phosphorus, and organic carbon (Philippot et al., 2023). Microorganisms in such environments depend heavily on efficient resource utilization and compete with one another for resources (Faust and Raes, 2012; Berry and Widder, 2014; Lima-Mendez et al., 2015). This scenario corresponds to the intricate microbial networks observed in CROWN S. Conversely, in high-nutrient soil environments (CROWN P), the greater ease with which microorganisms can access requisite nutrients results in a diminished impact of environmental factors on their community composition. This conclusion, however, does not imply an absence of a relationship between microorganisms and environmental factors in high-nutrient environments. Microorganisms under these conditions may display a higher degree of adaptability to resource availability that leads to a less pronounced response to environmental factors. Collectively, this analysis provided further evidence of the greater stability of the soil microorganisms in the *P. australis* wetland compared to the *S. alterniflora* wetland in this study.

The high correlation between soil microbial communities and ecosystem respiration (R_eco_) indicated a close association between changes in community composition and the potential for carbon degradation. As anticipated, predictive functions based on taxonomic analysis using the Faprotax and FunGuild databases revealed that the soil microorganisms in CROWN S had a significantly higher carbon decomposition potential (Supplementary Tables S11, S12) that aligned with the relatively high R_eco_ values in CROWN S (Table 1). Similarly, previous research has indicated that the invasion of *S. alterniflora* enhanced microbial carbon metabolism (Zhang et al., 2023). As illustrated in Figure 5C, experimental warming had a positive effect on the carbon degradation potential at both CROWN S and CROWN P site, suggesting that climate warming may exacerbate the decomposition of organic carbon in coastal wetland soils, as observed in other ecosystems (Giardina et al., 2014; Xue et al., 2016; Wang et al., 2021b), and consequently might reduce the carbon sequestration potential of coastal wetlands. Notably, the invasive *S. alterniflora* wetlands were more sensitive to warming than the native *P. australis* wetlands, with the response of carbon degradation potential was more pronounced in CROWN S (Figure 5C). As reported in our previous study, the carbon sequestration capacity of *S. alterniflora* wetlands decreases and the wetland may even become a carbon source under a warming scenario (Zhou P. et al., 2023). The implication is that *S. alterniflora* wetlands may release more CO_2_ into the atmosphere as the climate warms and exacerbate rather than mitigate the negative effects of climate change. This possibility underscores the importance of wetland management and conservation and particularly emphasizes the need to avoid indiscriminate use of *S. alterniflora* for coastal protection.

## 5 Conclusion

This study revealed that climate warming has a significant impact on the turnover rates of soil prokaryotic and fungal communities. The response of soil microbial network patterns to experimental warming differed between native *P. australis* wetlands and invasive *S. alterniflora* wetlands. Specifically, the network patterns in the *P. australis* wetland were found to be more stable under warming, while in the *S. alterniflora* wetland, they were less stable. The greater increase of the relative abundance of prokaryotic OTUs involved in carbon degradation in response to warming in the invasive *S. alterniflora* wetlands could lead to positive feedback and a release rather than uptake of CO_2_. We thus suggest that in the context of climate warming, *P. australis* wetlands exhibit greater adaptability and stability compared to *S. alterniflora* wetlands. The former are best suited to maintaining carbon sequestration functions in coastal wetlands. Overall, preserving native *P. australis* wetlands from *S. alterniflora* invasion could be crucial for mitigating the detrimental effects of warming-induced changes on ecosystem functions in China.

## Data availability statement

The datasets presented in this study can be found in online repositories. The names of the repository/repositories and accession number(s) can be found at: https://www.ncbi.nlm.nih.gov/, PRJNA1042794 and PRJNA1042799.

## Author contributions

LP: Conceptualization, Formal analysis, Investigation, Methodology, Visualization, Writing – original draft. SiY: Conceptualization, Funding acquisition, Project administration, Resources, Writing – review & editing. LX: Investigation, Supervision, Writing – review & editing. PZ: Formal analysis, Investigation, Writing – review & editing. LH: Investigation, Writing – review & editing. ShY: Investigation, Writing – review & editing. XD: Investigation, Writing – review & editing. HY: Investigation, Writing – review & editing. TD: Software, Writing – review & editing. EL: Writing – review & editing.

## Footnotes

^1^ www.majorbio.com

^2^ http://ieg4.rccc.ou.edu/mena/
