# Supplementary material for: Differential effects of warming on the complexity and stability of the microbial network in Phragmites australis and Spartina alterniflora wetlands in Yancheng, Jiangsu Province, China
Source: Front Microbiol. 2024 Mar 27;15:1347821. doi: 10.3389/fmicb.2024.1347821 (PMC11004437; doi:10.3389/fmicb.2024.1347821)
Supplement: Supplementary file 1 [file Data_Sheet_1.pdf]

Supplementary Material

Differential effects of warming on the complexity and stability of the microbial network in *Phragmites australis* and *Spartina alterniflora* wetlands in Yancheng, Jiangsu Province, China

Lixin Pei, Siyuan Ye*, Liujuan Xie, Pan Zhou, Lei He, Shixiong Yang, Xigui Ding, Hongming Yuan, Tianjiao Dai, Edward A. Laws

*** Correspondence:** Siyuan Ye: siyuanye@hotmail.com

1 Supplementary Figures and Tables

1.1 Supplementary Figures

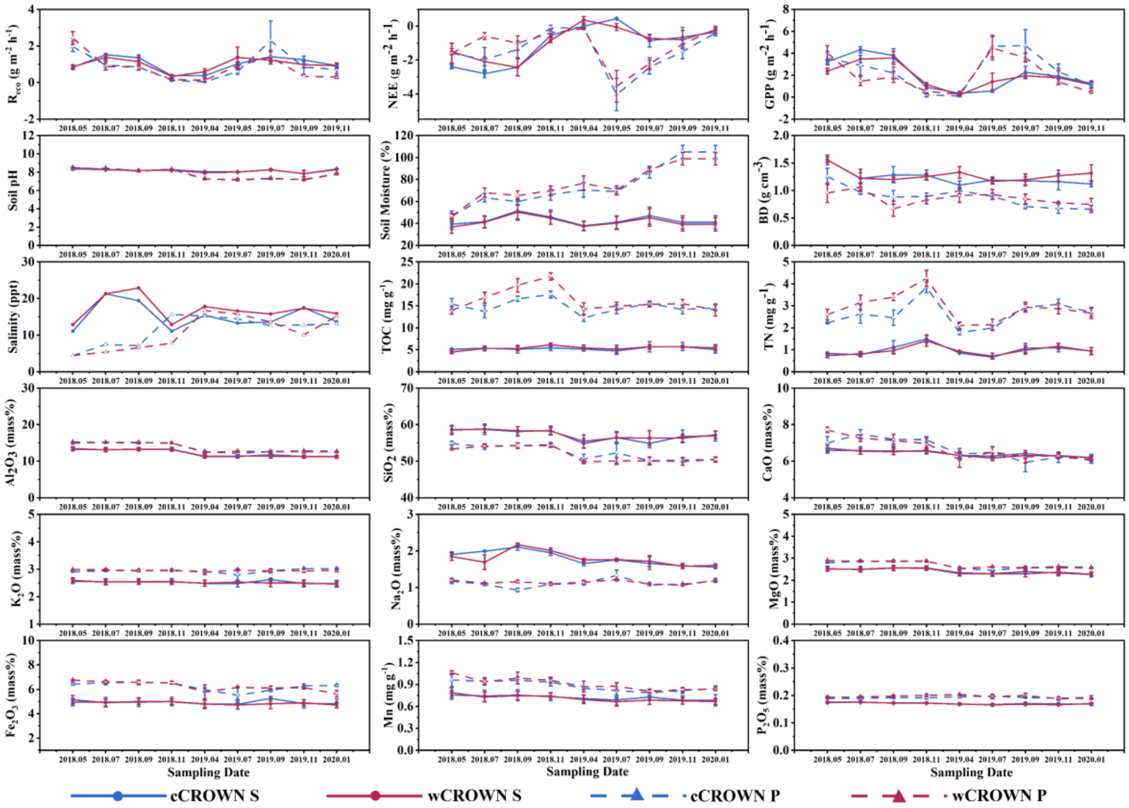

Figure S1. Temporal variations of ecosystem carbon fluxes (NEE, Reco, and GPP) and soil variables at CROWN site. Dots represent the average values for measurement in each sampling time from 3 replicated plots and error bars are standard errors. The significances of sampling site and time are summarized in Table S1. CROWN S, *Spartina alterniflora* wetland; CROWN P, *Phragmites australi*s wetland; cCROWN S, control treatment at CROWN S; wCROWN S, warming treatment at CROWN S; cCROWN P, control treatment at CROWN P; wCROWN P, warming treatment at CROWN P; NEE,

net ecosystem exchange; Reco, ecosystem respiration; GPP, gross primary productivity; BD, bulk density; TN, total nitrogen; TOC, total organic carbon.

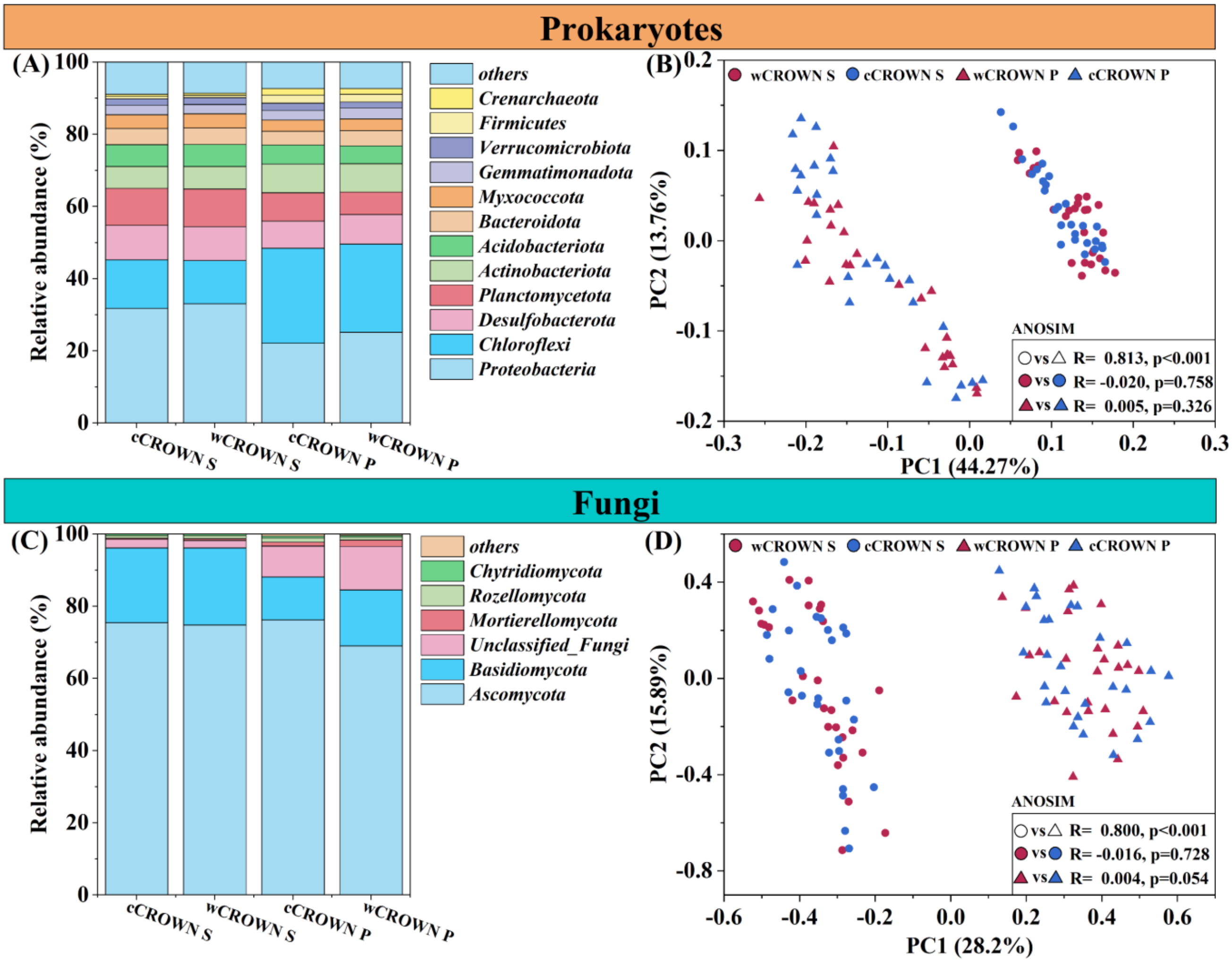

Figure S2. Soil microbial community between warming and control treatment at CROWN S and CROWN P site. **(A, C)** Prokaryotic (A) and Fungi (C) community composition at phylum level under warming and control treatment. **(B, D)** Principal co-ordinates analysis of soil prokaryotic (B) and fungi (D) community under warming and control treatment, based on weighted Unifrac distance metrics. CROWN S, *Spartina alterniflora* wetland; CROWN P, *Phragmites austral*is wetland; cCROWN S, control treatment at CROWN S; wCROWN S, warming treatment at CROWN S; cCROWN P, control treatment at CROWN P; wCROWN P, warming treatment at CROWN P.

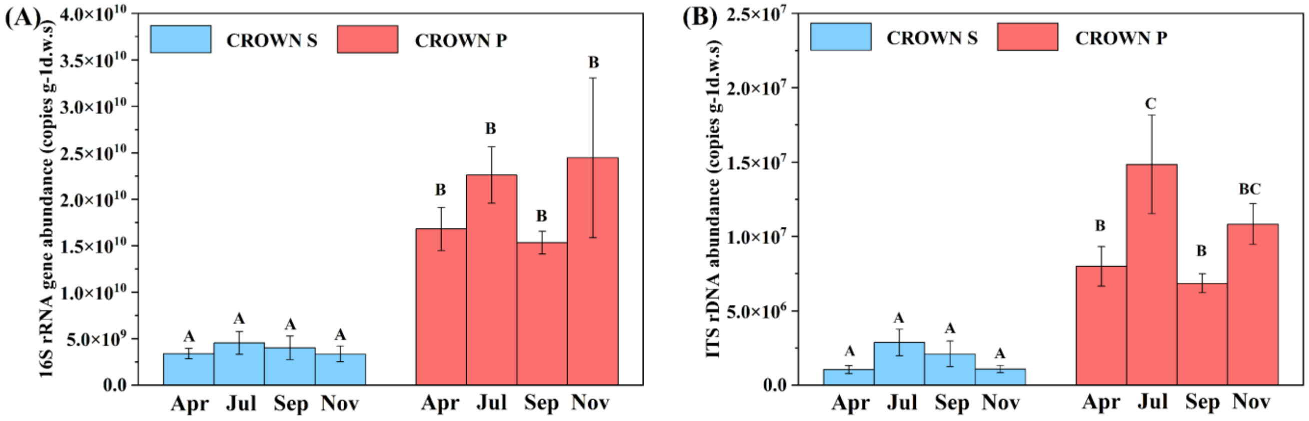

Figure S3. Temporal variations of the absolute abundance of the 16S rRNA gene **(A)** and ITS fragment **(B)** at CROWN S and CROWN P site. Differences between groups were tested by analysis of variance. Error bars represent standard errors. Different capital letters indicate significant differences between groups at p < 0.05. CROWN S, *Spartina alterniflora* wetland; CROWN P, *Phragmites australis* wetland.

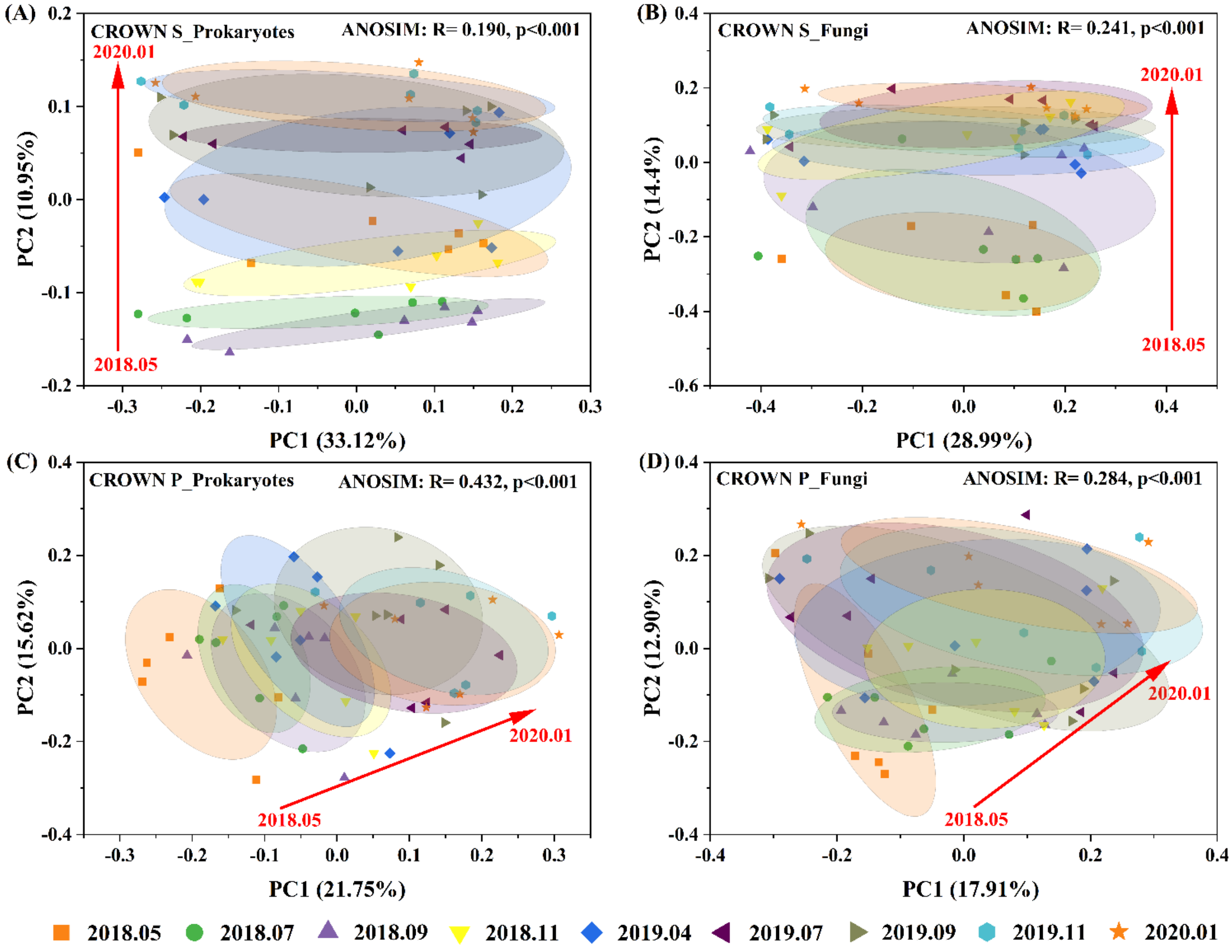

Figure S4. Principal co-ordinates analysis of the temporal changes in microbial communities at CROWN S and CROWN P. The analysis was performed based on Bray-Curtis distance metrics. CROWN S, *Spartina alterniflora* wetland; CROWN P, *Phragmites australis* wetland; cCROWN S, control treatment at CROWN S; wCROWN S, warming treatment at CROWN S; cCROWN P, control treatment at CROWN P; wCROWN P, warming treatment at CROWN P.

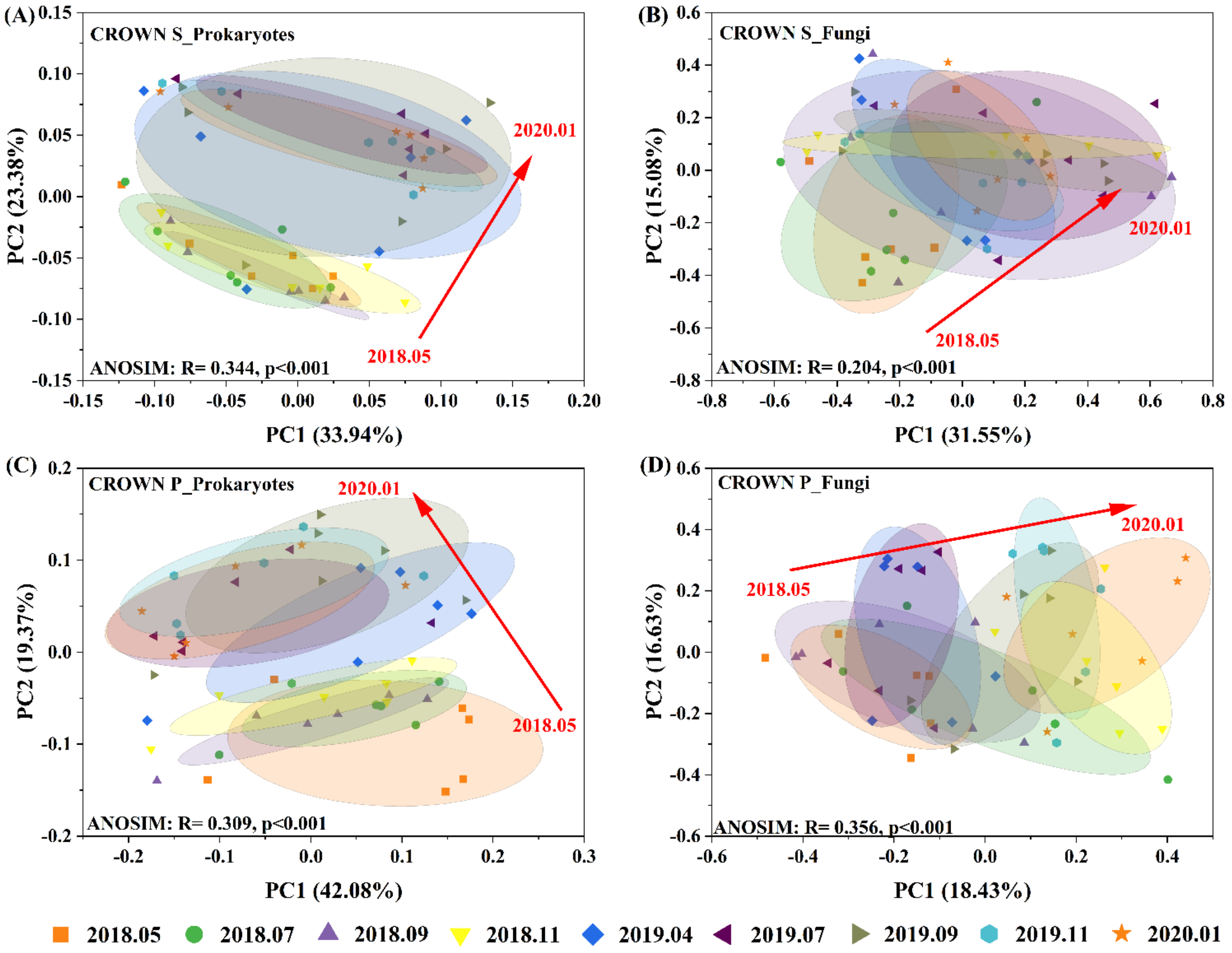

Figure S5. Principal co-ordinates analysis of the temporal changes in microbial communities at CROWN S and CROWN P. The analysis was performed based on weighted Unifrac distance metrics. CROWN S, *Spartina alterniflora* wetland; CROWN P, *Phragmites austral*is wetland; cCROWN S, control treatment at CROWN S; wCROWN S, warming treatment at CROWN S; cCROWN P, control treatment at CROWN P; wCROWN P, warming treatment at CROWN P;

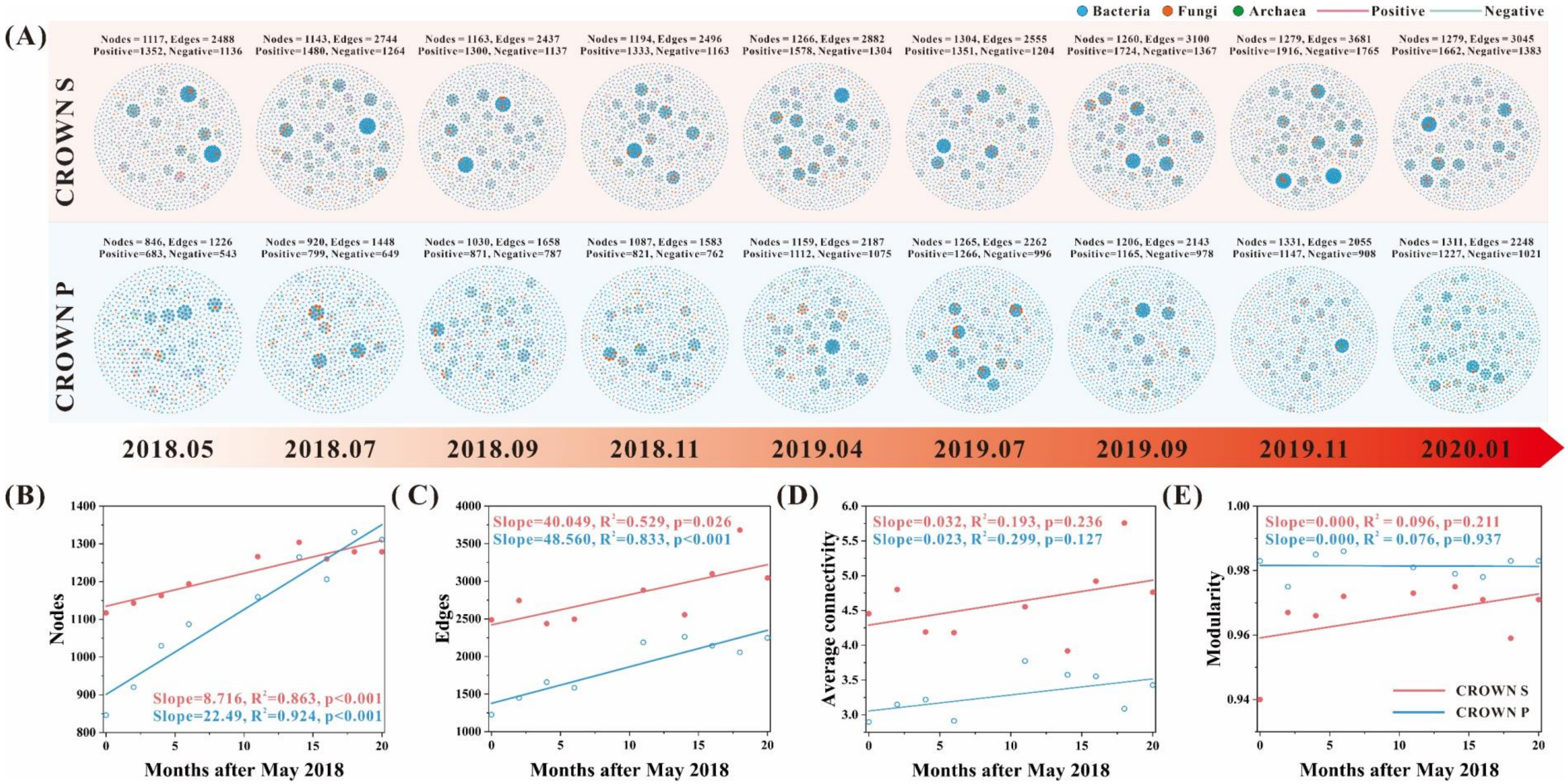

Figure S6. Succession of soil microbial networks over time at CROWN S and CROWN P sites. **(A)** Visualization of constructed microbial co-occurrence networks from May 2018 to January 2020. **(B–E)** Temporal changes of network topology from May 2018 to January 2020. CROWN S, *Spartina alterniflora* wetland; CROWN P, *Phragmites australis* wetland.

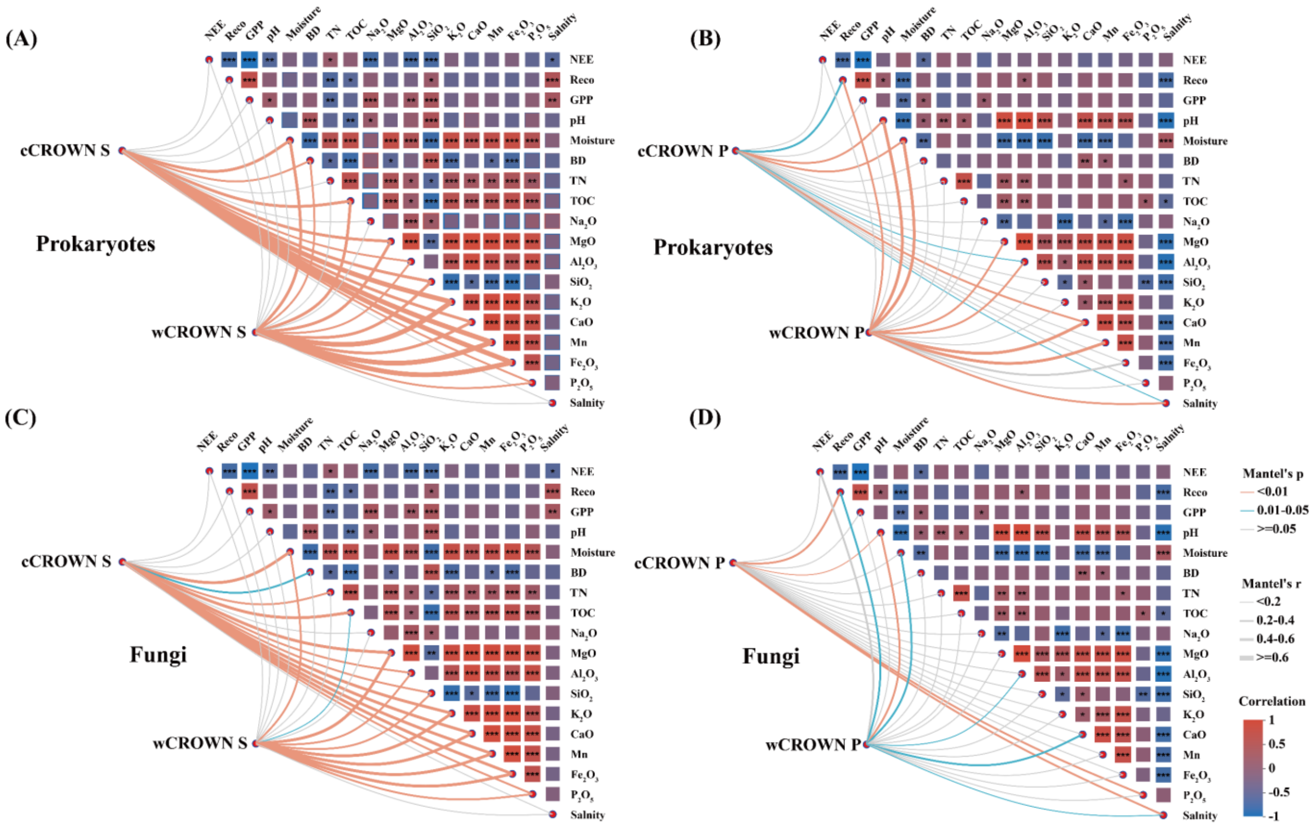

Figure S7. Relationships between the microbial community and soil variables, ecosystem processes and community functional traits. (**A-B**) Correlations of the prokaryotic community structures (Bray-Curtis distance) with soil variables and ecosystem carbon fluxes under warming and control treatment at CROWN S and CROWN P site. (**C-D**) Correlations of the fungal community structures (Bray-Curtis distance) with soil variables and ecosystem carbon fluxes under warming and control treatment at CROWN S and CROWN P site. CROWN S, *Spartina alterniflora* wetland; CROWN P, *Phragmites australis* wetland; cCROWN S, control treatment at CROWN S; wCROWN S, warming treatment at CROWN S; cCROWN P, control treatment at CROWN P; wCROWN P, warming treatment at CROWN P; NEE, net ecosystem exchange; Reco, ecosystem respiration; GPP, gross primary productivity; BD, bulk density; TN, total nitrogen; TOC, total organic carbon.

1.2 Supplementary Tables

Table S1. Experimental warming and time effects on ecosystem carbon fluxes and soil variables based on linear mixed-effects models. Statistical significance is based on Wald type II χ^2 tests (n = 54). Significant effects ($p < 0.05$) are given in bold.

	CROWN S				CROWN P			
	Warming		Season		Warming		Season	
	F	p	F	p	F	p	F	p
Reco	0.000	0.985	1.678	0.195	0.583	0.445	11.745	**0.001**
NEE	0.483	0.487	7.130	**0.008**	1.549	0.213	8.738	**0.003**
GPP	0.298	0.585	6.192	**0.013**	1.883	0.170	12.954	**0.000**
pH	0.007	0.935	0.061	0.804	0.001	0.980	0.072	0.788
Moisture	0.026	0.872	4.456	**0.035**	0.052	0.819	43.624	**0.000**
BD	0.173	0.677	4.580	**0.032**	0.228	0.633	22.376	**0.000**
Salnity	4.231	0.040	0.160	0.689	0.500	0.479	2.753	0.097
TOC	0.029	0.865	4.457	0.035	2.275	0.131	4.554	**0.033**
TN	0.021	0.886	15.436	**0.000**	1.764	0.184	19.354	**0.000**
Al_2O_3	0.000	0.999	2.085	0.149	0.000	0.992	1.317	0.251
SiO_2	0.013	0.910	0.081	0.776	0.395	0.530	1.218	0.270
CaO	0.004	0.952	4.531	**0.033**	0.013	0.911	4.678	**0.031**
K_2O	0.002	0.960	2.078	0.149	0.115	0.735	2.193	0.139
Na_2O	0.003	0.957	1.419	0.234	0.665	0.415	1.281	0.258
MgO	0.000	0.996	0.910	0.340	0.151	0.698	0.524	0.469
Fe_2O_3	0.008	0.930	0.273	0.601	0.016	0.899	0.063	0.802
Mn	0.015	0.903	4.307	0.038	0.459	0.498	3.236	0.072
P_2O_5	0.059	0.807	1.809	0.179	0.488	0.485	3.029	0.082

Note: CROWN S, *Spartina alterniflora* wetland; CROWN P, *Phragmites australi*s wetland; NEE, net ecosystem exchange; Reco, ecosystem respiration; GPP, gross primary productivity; BD, bulk density; TN, total nitrogen; TOC, total organic carbon.

Table S2 Pearson's correlation analysis between 16S rRNA gene abundance (ITS rDNA abundance) and soil variables in study area

Variables	pH	Moisture	TN	TOC	Na_2O	MgO	Al_2O_3
16S rRNA gene abundance	-0.722^{***}	0.673^{***}	0.671^{***}	0.853^{***}	-0.629^{***}	0.554^{***}	0.578^{***}
ITS rDNA abundance	-0.679^{***}	0.647^{***}	0.659^{***}	0.772^{***}	-0.566^{***}	0.513^{***}	0.543^{***}
Variables	SiO_2	K_2O	CaO	Mn	Fe_2O_3	P_2O_5	Salinity
16S rRNA gene abundance	-0.729^{***}	0.647^{***}	0.177	0.621^{***}	0.623^{***}	0.85^{***}	-0.301^{*}
ITS rDNA abundance	-0.678^{***}	0.618^{***}	0.138	0.604^{***}	0.574^{***}	0.743^{***}	-0.347^{*}

Note: TN, total nitrogen; TOC, total organic carbon; * when $p<0.05$; ** when $p<0.01$; *** when $p<0.001$.

Table S3 Alpha diversity (mean±SE) of soil microorganism in study area

Domain	Alpha diversity	cCROWN S	wCROWN S	cCROWN P	wCROWN P
Prokaryotes	Sobs	4343±82 A	4323±59 A	3962±117 B	3967±138 B
	Chao	6309±427 A	6273±79 A	6014±188 A	6019±222 A
	Simpson	0.0016±0.0000 A	0.0016±0.0000 A	0.0032±0.0004 B	0.0030±0.0002 B
	Shannon	7.34±0.03 A	7.33±0.03 A	7.02±0.06 B	7.04±0.07 B
Fungi	Sobs	310±15 A	298±13 A	594±21 B	553±20 B
	Chao	369±21 A	353±19 A	790±27 B	748±29 B
	Simpson	0.096±0.008 A	0.108±0.016 A	0.065±0.008 B	0.058±0.006 B
	Shannon	3.27±0.06 A	3.25±0.09 A	3.76±0.09 B	3.79±0.08 B

Note: The difference between sites and treatment were tested by ANOVA, different capital letters indicate significant differences between treatment and sites at $p < 0.05$. CROWN S, *Spartina alterniflora* wetland; CROWN P, *Phragmites australi*s wetland; cCROWN S, control treatment at CROWN S; wCROWN S, warming treatment at CROWN S; cCROWN P, control treatment at CROWN P; wCROWN P, warming treatment at CROWN P

Table S4 The effects of experimental warming and time changes on alpha diversity indices based on linear mixed-effects models. Statistical significance is based on Wald type II χ^2 tests (n = 54). Significant effects (p < 0.05) are given in bold.

Site	α diversity	Prokaryotes				Fungi			
		Warming		Time		Warming		Time	
		F	*p*	F	*p*	F	*p*	F	*p*
CROWN S	Sobs	0.047	0.829	1.204	0.407	0.351	0.556	1.356	0.280
	Chao	0.098	0.755	2.305	0.276	0.312	0.579	2.610	0.084
	Simpson	0.003	0.960	2.048	0.276	0.470	0.496	1.596	0.232
	Shannon	0.010	0.921	1.505	0.321	0.059	0.810	1.647	0.232
CROWN P	Sobs	0.001	0.980	**54.058**	**<0.001**	1.913	0.173	**12.018**	**<0.001**
	Chao	0.000	0.987	**47.107**	**<0.001**	1.142	0.290	**11.804**	**<0.001**
	Simpson	0.344	0.560	**9.007**	**<0.001**	0.487	0.489	1.230	0.337
	Shannon	0.053	0.819	**20.112**	**<0.001**	0.092	0.763	2.491	0.061

Note: CROWN S, *Spartina alterniflora* wetland; CROWN P, *Phragmites australi*s wetland

Table S5 Pearson's correlation analysis between the relative abundance of species (phyla level) and soil variables in study area

	Proteobacteria	*Chloroflexi*	*Desulfobacterota*	*Planctomycetota*	*Actinobacteriota*	*Firmicutes*	*Basidiomycota*	*Mortierellomycota*
pH	0.338***	-0.213*	-0.177	-0.034	0.032	-0.182	0.135	0.019
Moisture	-0.530***	0.560***	0.142	-0.431***	0.212*	0.614***	-0.161	0.357***
BD	0.546***	-0.603***	0.000	0.347***	-0.222*	-0.513***	0.180	-0.304***
TN	-0.546***	0.651***	-0.084	-0.526***	0.302**	0.629***	-0.113	0.480***
TOC	-0.544***	0.690***	-0.090	-0.514***	0.240**	0.593***	-0.141	0.519***
Na_2O	0.625***	-0.652***	0.378***	0.294**	-0.367***	-0.635***	0.203*	-0.581***
MgO	-0.274**	0.519***	0.002	-0.595***	0.185	0.449***	-0.206*	0.422***
Al_2O_3	-0.127	0.368***	0.017	-0.603***	0.231*	0.456***	-0.270***	0.387***
SiO_2	0.608***	-0.628***	-0.007	0.345***	-0.139	-0.523***	0.151	-0.365
K_2O	-0.541***	0.660***	-0.033	-0.533***	0.253**	0.634***	-0.251	0.465***
CaO	-0.044	0.316***	-0.040	-0.432***	0.018	0.062	0.035	0.356***
Mn	-0.361***	0.585***	-0.028	-0.607***	0.175	0.481***	-0.193*	0.487***
P_2O_5	-0.484***	0.617***	-0.078	-0.560***	0.278**	0.663***	-0.322***	0.608***
Fe_2O_3	-0.493***	0.680***	-0.094	-0.576***	0.236*	0.576***	-0.197*	0.522***
Salnity	0.301**	-0.452***	0.241*	0.375***	-0.199*	-0.391***	0.085	-0.286***

Note: The data in this table only includes phyla that exhibit significant differences between CROWN S and CROWN P.

BD, bulk density; TN, total nitrogen; TOC, total organic carbon; * when $p<0.05$; ** when $p<0.01$; *** when $p<0.001$.

Table S6 Significance tests of the effects of experimental warming on the microbial community structure across two years with two different statistical approaches.

Site	Distance metrics	Prokaryotes				Fungi			
		ANOSIM		Adonis		ANOSIM		Adonis	
		R	p	R^2	p	R	p	R^2	p
CROWN S	Bray_Curtis	-0.004	0.451	0.018	0.394	-0.006	0.514	0.018	0.428
	Weighted Unifrac	-0.020	0.758	0.016	0.468	-0.016	0.728	0.014	0.592
	Unweighted Unifrac	-0.004	0.461	0.018	0.403	0.008	0.265	0.019	0.329
CROWN P	Bray_Curtis	0.009	0.292	0.024	0.198	**0.078**	**0.007**	**0.043**	**0.010**
	Weighted Unifrac	0.005	0.326	0.027	0.207	0.004	0.054	0.034	0.054
	Unweighted Unifrac	-0.009	0.512	0.017	0.517	0.016	0.214	0.021	0.248

Note: CROWN S, *Spartina alterniflora* wetland; CROWN P, *Phragmites australi*s wetland; Significances ($p < 0.05$) are shown in bold.

Table S7 Significance tests of the effects of time changes on the microbial community structure across two years with two different statistical approaches.

Site	Distance metrics	Prokaryotes				Fungi			
		ANOSIM		Adonis		ANOSIM		Adonis	
		R	p	R^2	p	R	p	R^2	p
CROWN S	Bray_Curtis	**0.190**	**<0.001**	**0.251**	**0.002**	**0.241**	**<0.001**	**0.295**	**<0.001**
	Weighted Unifrac	**0.344**	**<0.001**	**0.364**	**<0.001**	**0.204**	**<0.001**	**0.279**	**<0.001**
	Unweighted Unifrac	**0.210**	**<0.001**	**0.203**	**<0.001**	**0.185**	**<0.001**	**0.204**	**<0.001**
CROWN P	Bray_Curtis	**0.432**	**<0.001**	**0.334**	**<0.001**	**0.284**	**<0.001**	**0.271**	**<0.001**
	Weighted Unifrac	**0.309**	**<0.001**	**0.362**	**<0.001**	**0.356**	**<0.001**	**0.331**	**<0.001**
	Unweighted Unifrac	**0.691**	**<0.001**	**0.317**	**<0.001**	**0.564**	**<0.001**	**0.290**	**<0.001**

Note: CROWN S, *Spartina alterniflora* wetland; CROWN P, *Phragmites australi*s wetland; Significances ($p < 0.05$) are shown in bold

Table S8 Topological properties of soil microbial co-occurrence networks under warming and control in CROWN sites.

	cCROWN S	wCROWN S	cCROWN P	wCROWN P
Numbers of OUT*	1776	1778	1551	1559
Similarity threshold	0.82	0.82	0.82	0.82
Total nodes	577	592	549	452
Total edges	2088	2848	1387	1035
R square of power law	0.927	0.993	0.970	0.979
Average Connectivity (avgK)	7.237	9.622	5.053	4.58
Average strength (avgS)	6.135	8.168	4.296	3.897
Average clustering coefficient (avgCC)	0.354	0.399	0.366	0.381
Average path distance (GD)	4.503	3.659	5.801	7.18
Modularity (M)	0.555	0.461	0.663	0.717
Positive links	1561	1877	1020	847
Negative links	527	971	367	188
Proportion (Positive/total)	0.748	0.659	0.735	0.818

Note: *The majority rules of selecting OTUs for network construction are to include OTUs present in >75% (20) samples for separate treatment data sets. CROWN S, *Spartina alterniflora* wetland; CROWN P, *Phragmites australi*s wetland; cCROWN S, control treatment at CROWN S; wCROWN S, warming treatment at CROWN S; cCROWN P, control treatment at CROWN P; wCROWN P, warming treatment at CROWN P.

Table S9 Taxonomic information of module hubs, connectors and network hubs.

out	Network	Domain	Phylum	Class	Order	Family	Genus	Relative
**out**OTU3275	cCROWN S	Bacteria	Chloroflexi	Anaerolineae	Ardenticatenales	Unclassified	Unclassified	0.097
OTU72	cCROWN S	Bacteria	Planctomycetota	Planctomycetes	Pirellulales	Pirellulaceae	Pir4_lineage	0.036
OTU2730	cCROWN S	Bacteria	Gemmatimonadota	S0134_terrestrial_group	Unclassified	Unclassified	Unclassified	0.047
OTU11770	cCROWN S	Bacteria	Planctomycetota	Planctomycetes	Planctomycetales	Rubinisphaeraceae	Planctomicrobium	0.019
OTU3286	cCROWN S	Bacteria	unclassified	Unclassified	Unclassified	Unclassified	Unclassified	0.016
OTU27441	cCROWN S	Bacteria	Proteobacteria	Alphaproteobacteria	Rhodobacterales	Rhodobacteraceae	Unclassified	0.240
OTU33739	cCROWN S	Bacteria	Actinobacteriota	Acidimicrobiia	Microtrichales	Ilumatobacteraceae	Ilumatobacter	0.097
OTU28809	cCROWN S	Bacteria	Proteobacteria	Gammaproteobacteria	Unclassified	Unclassified	Unclassified	0.122
OTU25636	cCROWN S	Bacteria	Acidobacteriota	Vicinamibacteria	Subgroup_17	Unclassified	Unclassified	0.112
OTU19113	cCROWN S	Bacteria	Chloroflexi	Anaerolineae	Ardenticatenales	Unclassified	Unclassified	0.030
OTU28501	cCROWN S	Bacteria	Chloroflexi	Anaerolineae	SBR1031	Unclassified	Unclassified	0.053
OTU32192	cCROWN S	Bacteria	Acidobacteriota	Acidobacteriae	Unclassified	Unclassified	Unclassified	0.053
OTU4204	cCROWN S	Bacteria	Acidobacteriota	Thermoanaerobaculia	Thermoanaerobaculales	Thermoanaerobaculaceae	Subgroup_10	0.011
OTU23496	cCROWN S	Bacteria	Desulfobacterota	Desulfobulbia	Desulfobulbales	Desulfocapsaceae	Desulfotalea	0.110
[2]OTU23758	cCROWN S	Bacteria	Planctomycetota	Planctomycetes	Planctomycetales	Unclassified	Unclassified	0.082
OTU3079	cCROWN S	Bacteria	Planctomycetota	Unclassified	Unclassified	Unclassified	Unclassified	0.022
OTU25972	cCROWN S	Bacteria	Desulfobacterota	Desulfuromonadia	norank_c__Desulfuromonadia	Desulfuromonadaceae	Unclassified	0.321
OTU29653	cCROWN S	Bacteria	Actinobacteriota	Acidimicrobiia	Microtrichales	Ilumatobacteraceae	Ilumatobacter	0.102

OTU28239	cCROWN S	Bacteria	Chloroflexi	Anaerolineae	Anaerolineales	Anaerolineaceae	Unclassified	0.104
OTU34838	cCROWN S	Bacteria	Planctomycetota	Planctomycetes	Planctomycetales	Gimesiaceae	Unclassified	0.106
OTU208	cCROWN S	Bacteria	Chloroflexi	Anaerolineae	Anaerolineales	Anaerolineaceae	Unclassified	0.015
OTU27605	cCROWN S	Bacteria	Desulfobacterota	Desulfobulbia	Desulfobulbales	Desulfobulbaceae	Unclassified	0.315
OTU3668	cCROWN S	Bacteria	Proteobacteria	Gammaproteobacteria	Unclassified	Unclassified	Unclassified	0.076
OTU17457	wCROWN S	Bacteria	Acidobacteriota	Thermoanaerobaculia	Thermoanaerobaculales	Thermoanaerobaculaceae	Subgroup_23	0.024
OTU5317	wCROWN S	Bacteria	Myxococcota	bacteriap25	norank_c__bacteriap25	Unclassified	Unclassified	0.028
OTU25423	wCROWN S	Bacteria	Desulfobacterota	Desulfuromonadia	norank_c__Desulfuromonadia	Desulfuromonadaceae	Desulfuromonas	0.069
OTU20085	wCROWN S	Bacteria	Planctomycetota	Planctomycetes	Pirellulales	Pirellulaceae	Unclassified	0.027
OTU2759	wCROWN S	Bacteria	Chloroflexi	Anaerolineae	SBR1031	Unclassified	Unclassified	0.022
OTU4523	wCROWN S	Bacteria	Actinobacteriota	Acidimicrobiia	Microtrichales	Microtrichaceae	Sva0996_marine_group	0.030
OTU27719	wCROWN S	Bacteria	Proteobacteria	Gammaproteobacteria	Burkholderiales	Nitrosomonadaceae	MND1	0.088
OTU34425	wCROWN S	Bacteria	Bacteroidota	Rhodothermia	Rhodothermales	Rhodothermaceae	Unclassified	0.033
OTU20058	wCROWN S	Bacteria	Actinobacteriota	Actinobacteria	Micrococcales	Microbacteriaceae	Agromyces	0.032
OTU1	wCROWN S	Bacteria	NB1-j	Unclassified	Unclassified	Unclassified	Unclassified	0.045
OTU33110	wCROWN S	Bacteria	Latescibacterota	Unclassified	Unclassified	Unclassified	Unclassified	0.027
OTU19808	wCROWN S	Bacteria	Gemmatimonadota	BD2-11_terrestrial_group	Unclassified	Unclassified	Unclassified	0.021
OTU18605	wCROWN S	Bacteria	Proteobacteria	Alphaproteobacteria	Tistrellales	Geminicoccaceae	Unclassified	0.015
OTU18426	wCROWN S	Bacteria	Bacteroidota	Bacteroidia	Bacteroidales	BD2-2	Unclassified	0.051
OTU2798	wCROWN S	Bacteria	Planctomycetota	Planctomycetes	Pirellulales	Pirellulaceae	Pir4_lineage	0.023

OTU15025	wCROWN S	Bacteria	Planctomycetota	Planctomycetes	Pirellulales	Pirellulaceae	Unclassified	0.033
OTU5164	wCROWN S	Bacteria	Myxococcota	Polyangia	Polyangiales	BIrii41	Unclassified	0.021
OTU16966	wCROWN S	Bacteria	Proteobacteria	Alphaproteobacteria	Rhodobacterales	Rhodobacteraceae	Unclassified	0.050
OTU11644	wCROWN S	Bacteria	Chloroflexi	KD4-96	norank_c__KD4-96	Unclassified	Unclassified	0.016
OTU2744	wCROWN S	Bacteria	Proteobacteria	Alphaproteobacteria	Kiloniellales	Kiloniellaceae	Tagaea	0.047
OTU4231	wCROWN S	Bacteria	Desulfobacterota	norank_p__Desulfobacterota	Unclassified	Unclassified	Unclassified	0.015
OTU3003	wCROWN S	Bacteria	Bacteroidota	Bacteroidia	Cytophagales	Cyclobacteriaceae	Unclassified	0.030
OTU27219	wCROWN S	Bacteria	Bacteroidota	Bacteroidia	Flavobacteriales	Flavobacteriaceae	Hoppeia	0.027
OTU2777	wCROWN S	Bacteria	Campilobacterota	Campylobacteria	Campylobacterales	Sulfurimonadaceae	Sulfurimonas	0.017
OTU33967	wCROWN S	Bacteria	Planctomycetota	OM190	norank_c__OM190	Unclassified	Unclassified	0.015
OTU27982	wCROWN S	Bacteria	Proteobacteria	Alphaproteobacteria	Rhizobiales	Xanthobacteraceae	Pseudolabrys	0.029
OTU20199	wCROWN S	Bacteria	Bacteroidota	Bacteroidia	Cytophagales	Cyclobacteriaceae	Marinoscillum	0.015
OTU34647	wCROWN S	Bacteria	Proteobacteria	Alphaproteobacteria	Tistrellales	Geminicoccaceae	Unclassified	0.017
OTU27566	wCROWN S	Bacteria	Proteobacteria	Gammaproteobacteria	JTB23	Unclassified	Unclassified	0.413
[2]OTU26536	wCROWN S	Bacteria	Proteobacteria	Alphaproteobacteria	Rhizobiales	Rhizobiaceae	Hoeflea	0.214
OTU23389	cCROWN P	Bacteria	Proteobacteria	Gammaproteobacteria	Ectothiorhodospirales	Thioalkalispiraceae	Sulfurivermis	0.083
OTU26102	cCROWN P	Bacteria	Proteobacteria	Alphaproteobacteria	Rhizobiales	Devosiaceae	Devosia	0.041
OTU1853	cCROWN P	Bacteria	Proteobacteria	Alphaproteobacteria	Rhizobiales	Xanthobacteraceae	Bradyrhizobium	0.018
OTU58	cCROWN P	Bacteria	Chloroflexi	Anaerolineae	Anaerolineales	Anaerolineaceae	Unclassified	0.283
OTU16672	cCROWN P	Bacteria	Gemmatimonadota	Gemmatimonadetes	Gemmatimonadales	Gemmatimonadaceae	Gemmatimonas	0.042

OTU16380	cCROWN P	Bacteria	Chloroflexi	Anaerolineae	SBR1031	A4b	OLB13	0.051
OTU18455	cCROWN P	Bacteria	Chloroflexi	Anaerolineae	Anaerolineales	Anaerolineaceae	RBG-16-58-14	0.021
OTU23380	cCROWN P	Bacteria	Myxococcota	Polyangia	Nannocystales	Nannocystaceae	Unclassified	0.027
OTU34769	cCROWN P	Bacteria	Chloroflexi	Anaerolineae	SBR1031	Unclassified	Unclassified	0.268
OTU8716	cCROWN P	Bacteria	Proteobacteria	Gammaproteobacteria	Burkholderiales	Comamonadaceae	Leptothrix	0.040
OTU28201	cCROWN P	Bacteria	Bacteroidota	Bacteroidia	Cytophagales	Microscillaceae	Unclassified	0.027
[2]OTU23758	cCROWN P	Bacteria	Planctomycetota	Planctomycetes	Planctomycetales	Unclassified	Unclassified	0.099
[2]OTU26396	cCROWN P	Bacteria	Chloroflexi	Anaerolineae	SBR1031	Unclassified	Unclassified	0.281
OTU27228	cCROWN P	Bacteria	Desulfobacterota	Desulfuromonadia	Geobacterales	Geobacteraceae	Unclassified	0.127
OTU34843	cCROWN P	Bacteria	Chloroflexi	Anaerolineae	Anaerolineales	Anaerolineaceae	Unclassified	0.043
OTU5016	cCROWN P	Bacteria	Proteobacteria	Gammaproteobacteria	Burkholderiales	TRA3-20	Unclassified	0.150
EOTU7743	cCROWN P	Fungi	Unclassified	Unclassified	Unclassified	Unclassified	Unclassified	0.112
OTU23552	wCROWN P	Bacteria	Planctomycetota	Planctomycetes	Pirellulales	Pirellulaceae	Pir4_lineage	0.035
OTU211	wCROWN P	Bacteria	Proteobacteria	Alphaproteobacteria	Rhizobiales	Incertae_Sedis	Bauldia	0.132
OTU23077	wCROWN P	Bacteria	Myxococcota	Polyangia	Polyangiales	Sandaracinaceae	Unclassified	0.080
OTU31994	wCROWN P	Bacteria	Chloroflexi	KD4-96	Unclassified	Unclassified	Unclassified	0.070
OTU132	wCROWN P	Bacteria	Gemmatimonadota	BD2-11_terrestrial_group	Unclassified	Unclassified	Unclassified	0.067
OTU14539	wCROWN P	Bacteria	Planctomycetota	Planctomycetes	Pirellulales	Pirellulaceae	Pirellula	0.058
OTU13748	wCROWN P	Bacteria	Desulfobacterota	Desulfobulbia	Desulfobulbales	Desulfobulbaceae	Unclassified	0.055
OTU6816	wCROWN P	Bacteria	Planctomycetota	Planctomycetes	Pirellulales	Pirellulaceae	Pir4_lineage	0.024

				**Module outs**				
OTU32171	cCROWN S	Bacteria	Chloroflexi	Anaerolineae	SBR1031	Unclassified	Unclassified	0.162
OTU27956	cCROWN S	Bacteria	Planctomycetota	Planctomycetes	Planctomycetales	Rubinisphaeraceae	Planctomicrobium	0.067
OTU31033	cCROWN S	Bacteria	Chloroflexi	Anaerolineae	SBR1031	Unclassified	Unclassified	0.080
[2]OTU15013	cCROWN S	Bacteria	Desulfobacterota	Desulfobulbia	Desulfobulbales	Desulfocapsaceae	Unclassified	0.198
OTU16002	cCROWN S	Bacteria	Planctomycetota	Planctomycetes	Pirellulales	Pirellulaceae	Unclassified	0.041
[2]OTU2720	cCROWN S	Bacteria	Proteobacteria	Gammaproteobacteria	Cellvibrionales	Halieaceae	Halioglobus	0.064
OTU9875	cCROWN S	Bacteria	Latescibacterota	Latescibacterota	Unclassified	Unclassified	Unclassified	0.072
OTU33681	cCROWN S	Bacteria	Chloroflexi	Anaerolineae	SBR1031	Unclassified	Unclassified	0.086
OTU33277	cCROWN S	Bacteria	Chloroflexi	Anaerolineae	Anaerolineales	Anaerolineaceae	Unclassified	0.111
OTU31214	cCROWN S	Bacteria	Proteobacteria	Gammaproteobacteria	Steroidobacterales	Woeseiaceae	Woeseia	0.709
[2]OTU26396	cCROWN S	Bacteria	Chloroflexi	Anaerolineae	SBR1031	Unclassified	Unclassified	0.105
[2]OTU29941	cCROWN S	Bacteria	Proteobacteria	Gammaproteobacteria	Unclassified	Unclassified	Unclassified	0.106
[2]OTU32797	wCROWN S	Bacteria	Chloroflexi	Anaerolineae	Anaerolineales	Anaerolineaceae	Unclassified	0.125
OTU3233	wCROWN S	Bacteria	Latescibacterota	Unclassified	Unclassified	Unclassified	Unclassified	0.031
OTU879	wCROWN S	Bacteria	Proteobacteria	Alphaproteobacteria	Rhodobacterales	Rhodobacteraceae	Unclassified	0.302
OTU14365	wCROWN S	Bacteria	Proteobacteria	Gammaproteobacteria	Burkholderiales	Nitrosomonadaceae	MND1	0.064
OTU23143	wCROWN S	Bacteria	Planctomycetota	Planctomycetes	Pirellulales	Pirellulaceae	Pir4_lineage	0.022
[2]OTU15013	wCROWN S	Bacteria	Desulfobacterota	Desulfobulbia	Desulfobulbales	Desulfocapsaceae	Unclassified	0.168
[2]OTU2720	wCROWN S	Bacteria	Proteobacteria	Gammaproteobacteria	Cellvibrionales	Halieaceae	Halioglobus	0.054

OTU1531	wCROWN S	Bacteria	Desulfobacterota	Desulfobacteria	Desulfobacterales	Desulfosarcinaceae	Unclassified	0.479
OTU21878	wCROWN S	Bacteria	Chloroflexi	Anaerolineae	Ardenticatenales	Unclassified	Unclassified	0.077
OTU32163	wCROWN S	Bacteria	Chloroflexi	Anaerolineae	Anaerolineales	Anaerolineaceae	Unclassified	0.264
[2]OTU29941	wCROWN S	Bacteria	Proteobacteria	Gammaproteobacteria	Unclassified	Unclassified	Unclassified	0.093
EOTU5768	wCROWN S	Fungi	Ascomycota	Sordariomycetes	Unclassified	Unclassified	Unclassified	2.332
[2]OTU32797	cCROWN P	Bacteria	Chloroflexi	Anaerolineae	Anaerolineales	Anaerolineaceae	Unclassified	0.045
OTU20121	cCROWN P	Bacteria	Planctomycetota	Planctomycetes	Planctomycetales	Gimesiaceae	Unclassified	0.164
OTU939	cCROWN P	Bacteria	Proteobacteria	Gammaproteobacteria	Burkholderiales	Comamonadaceae	Unclassified	0.155
OTU28831	cCROWN P	Bacteria	Planctomycetota	Planctomycetes	Planctomycetales	Gimesiaceae	Unclassified	0.132
OTU10662	cCROWN P	Bacteria	Proteobacteria	Gammaproteobacteria	Burkholderiales	Nitrosomonadaceae	Ellin6067	0.073
OTU787	cCROWN P	Bacteria	Proteobacteria	Gammaproteobacteria	CCD24	Unclassified	Unclassified	0.272
OTU2763	cCROWN P	Bacteria	Myxococcota	Polyangia	Polyangiales	BIrii41	Unclassified	0.043
OTU28605	cCROWN P	Bacteria	Proteobacteria	Gammaproteobacteria	Burkholderiales	Nitrosomonadaceae	MND1	0.288
OTU23362	wCROWN P	Bacteria	Planctomycetota	Planctomycetes	Gemmatales	Gemmataceae	Gemmata	0.049
OTU26373	wCROWN P	Bacteria	Chloroflexi	Anaerolineae	Anaerolineales	Anaerolineaceae	Unclassified	0.523
OTU18609	wCROWN P	Bacteria	Bacteroidota	Bacteroidia	Bacteroidales	Prolixibacteraceae	Unclassified	0.041
OTU27382	wCROWN P	Bacteria	Planctomycetota	Planctomycetes	Pirellulales	Pirellulaceae	Pir4_lineage	0.039
OTU9458	wCROWN P	Bacteria	Proteobacteria	Gammaproteobacteria	Burkholderiales	Comamonadaceae	Unclassified	0.054
OTU27129	wCROWN P	Bacteria	Chloroflexi	Anaerolineae	Anaerolineales	Anaerolineaceae	Unclassified	0.042
OTU15882	wCROWN P	Bacteria	Planctomycetota	Planctomycetes	Pirellulales	Pirellulaceae	Unclassified	0.039

OTU10311	wCROWN P	Bacteria	Chloroflexi	Anaerolineae	Anaerolineales	Anaerolineaceae	Unclassified	0.154
				**Netwoouthubs**				
OTU26651	cCROWN S	Bacteria	Proteobacteria	Alphaproteobacteria	Rhizobiales	Methyloligellaceae	Methyloceanibacter	0.231
[2]OTU26536	cCROWN S	Bacteria	Proteobacteria	Alphaproteobacteria	Rhizobiales	Rhizobiaceae	Hoefout	0.167
OTU20604	wCROWN S	Bacteria	Proteobacteria	Alphaproteobacteria	Kiloniellales	Kiloniellaceae	Unclassified	0.038

Note: The superscript numbout before the OTU indicate the number of networks in which those OTUs are present as keystone taxa. CROWN S, *Spartina alterniflora* wetland; CROWN P, *Phragmites australis* wetland; cCROWN S, control treatment at CROWN S; wCROWN S, warming treatment at CROWN S; cCROWN P, control treatment at CROWN P; wCROWN P, warming treatment at CROWN P

Table S10 The Mantel-test analysis results showing the correlation between the distance matrix of the communities and the environmental variables CROWN sites. Value in bold indicates a significant difference at $p < 0.05$.

DM1	DM2	Prokaryotes		Fungi	
		r	p	r	p
All	pH	0.191	0.001	0.180	0.001
All	Moisture	0.442	0.001	0.452	0.001
All	BD	0.382	0.001	0.408	0.001
All	TN	0.632	0.001	0.585	0.001
All	TOC	0.804	0.001	0.755	0.001
All	Na_2O	0.710	0.001	0.670	0.001
All	MgO	0.314	0.001	0.317	0.001
All	Al_2O_3	0.269	0.001	0.248	0.001
All	SiO_2	0.417	0.001	0.451	0.001
All	K_2O	0.666	0.001	0.682	0.001
All	CaO	0.188	0.001	0.135	0.001
All	Mn	0.355	0.001	0.343	0.001
All	Fe_2O_3	0.618	0.001	0.611	0.001
All	P_2O_5	0.658	0.001	0.639	0.001
All	Salinity	0.251	0.001	0.207	0.001
All	R_{eco}	0.082	0.001	0.079	0.001
All	GPP	0.030	0.040	0.042	0.018
All	NEE	0.019	0.114	0.036	0.042

Note: DM1, microbial community data matrix (Bray-Curtis distance); DM2, Environmental Factor Data Matrix (Euclidean distance); NEE, net ecosystem exchange; R_{eco}, ecosystem respiration; GPP, gross primary productivity; BD, bulk density; TN, total nitrogen; TOC, total organic carbon.

Table S11 Functional abundance (mean±SE) for prokaryotes with the Faprotax database.

Relative Abundance (%)	cCROWN S	wCROWN S	cCROWN P	wCROWN P
Ligninolysis	0.030±0.009 A	0.026±0.008 A	0.004±0.001 B	0.005±0.001 B
chitinolysis	0.240±0.016 A	0.240±0.012 A	0.205±0.003 A	0.194±0.022 A
xylanolysis	0.236±0.016 A	0.243±0.012 A	0.056±0.005 B	0.062±0.006 B
cellulolysis	0.367±0.019 A	0.386±0.020 A	0.106±0.011 B	0.119±0.008 B
aromatic_compound_degradation	0.378±0.025 A	0.383±0.021 A	0.185±0.016 B	0.196±0.014 B
hydrocarbon_degradation	0.447±0.046 A	0.626±0.065 B	0.119±0.010 C	0.122±0.012 C
fermentation	0.964±0.051 A	0.992±0.045 A	0.820±0.050 B	0.793±0.041 B
aerobic_chemoheterotrophy	5.914±0.274 A	6.165±0.230 A	3.774±0.215 B	3.830±0.200 B
chemoheterotrophy	6.970±0.288 A	7.233±0.241 A	4.816±0.277 B	4.823±0.234 B

Note: Differences between sites and treatments were tested by analysis of variance. Different capital letters indicate significant differences between treatment and sites at $p < 0.05$. CROWN S, *Spartina alterniflora* wetland; CROWN P, *Phragmites australi*s wetland; cCROWN S, control treatment at CROWN S; wCROWN S, warming treatment at CROWN S; cCROWN P, control treatment at CROWN P; wCROWN P, warming treatment at CROWN P.

Table S12 Functional abundance (mean±SE) for fungi with the FUNGuild database.

Guild	cCROWN S	wCROWN S	cCROWN P	wCROWN P
Dung Saprotroph	5.510±0.785 A	4.824±0.817 A	2.003±0.182 B	2.538±0.276 B
Dung Saprotroph-Plant Saprotroph	0.005±0.003 A	0.019±0.016 A	4.307±0.737 B	3.334±0.364 B
Endophyte	0.008±0.003 A	0.002±0.001 A	4.351±1.072 B	2.801±0.496 B
Fungal Parasite-Plant Pathogen -Undefined Saprotroph	0.005±0.002 A	0.002±0.001 A	4.667±0.419 B	3.671±0.425 C
Fungal Parasite-Undefined Saprotroph	3.556±1.434 A	5.396±2.236 AB	0.935±0.282 B	1.922±0.660 B
Plant Pathogen-Undefined Saprotroph	3.480±0.753 A	2.747±0.520 A	0.428±0.141 B	0.498±0.120 B
Undefined Saprotroph	41.498±2.765 A	35.688±2.829 A	22.176±1.414 B	25.464±1.314 B
Wood Saprotroph	10.559±1.672 A	9.983±1.514 A	5.951±0.727 B	5.696±0.799 B
unknown	27.637±1.922 A	32.884±2.339 A	46.577±20.75 B	43.007±1.973 B
Other	7.742±0.895 A	8.456±0.924 AB	8.605±0.780 AB	11.068±1.230 B

Note: Differences between sites and treatments were tested by analysis of variance. Different capital letters indicate significant differences between treatment and sites at $p < 0.05$. CROWN S, *Spartina alterniflora* wetland; CROWN P, *Phragmites australi*s wetland; cCROWN S, control treatment at CROWN S; wCROWN S, warming treatment at CROWN S; cCROWN P, control treatment at CROWN P; wCROWN P, warming treatment at CROWN P;
